# Supplementary material for: Phosphorylation-driven epichaperome assembly is a regulator of cellular adaptability and proliferation
Source: Nat Commun. 2024 Oct 16;15:8912. doi: 10.1038/s41467-024-53178-5 (PMC11484706; doi:10.1038/s41467-024-53178-5)
Supplement: Supplementary file 1 — Supplementary Information [file 41467_2024_53178_MOESM1_ESM.pdf]

Supplementary Information

Phosphorylation-driven epichaperome assembly is a regulator of cellular adaptability and proliferation

Roychowdhury, McNutt, Pasala et al.

Contains:

Supplementary Figures 1 through 15

Supplementary Tables 1 through 5

Supplementary Notes 1: synthetic procedures

Supplementary Figure 1

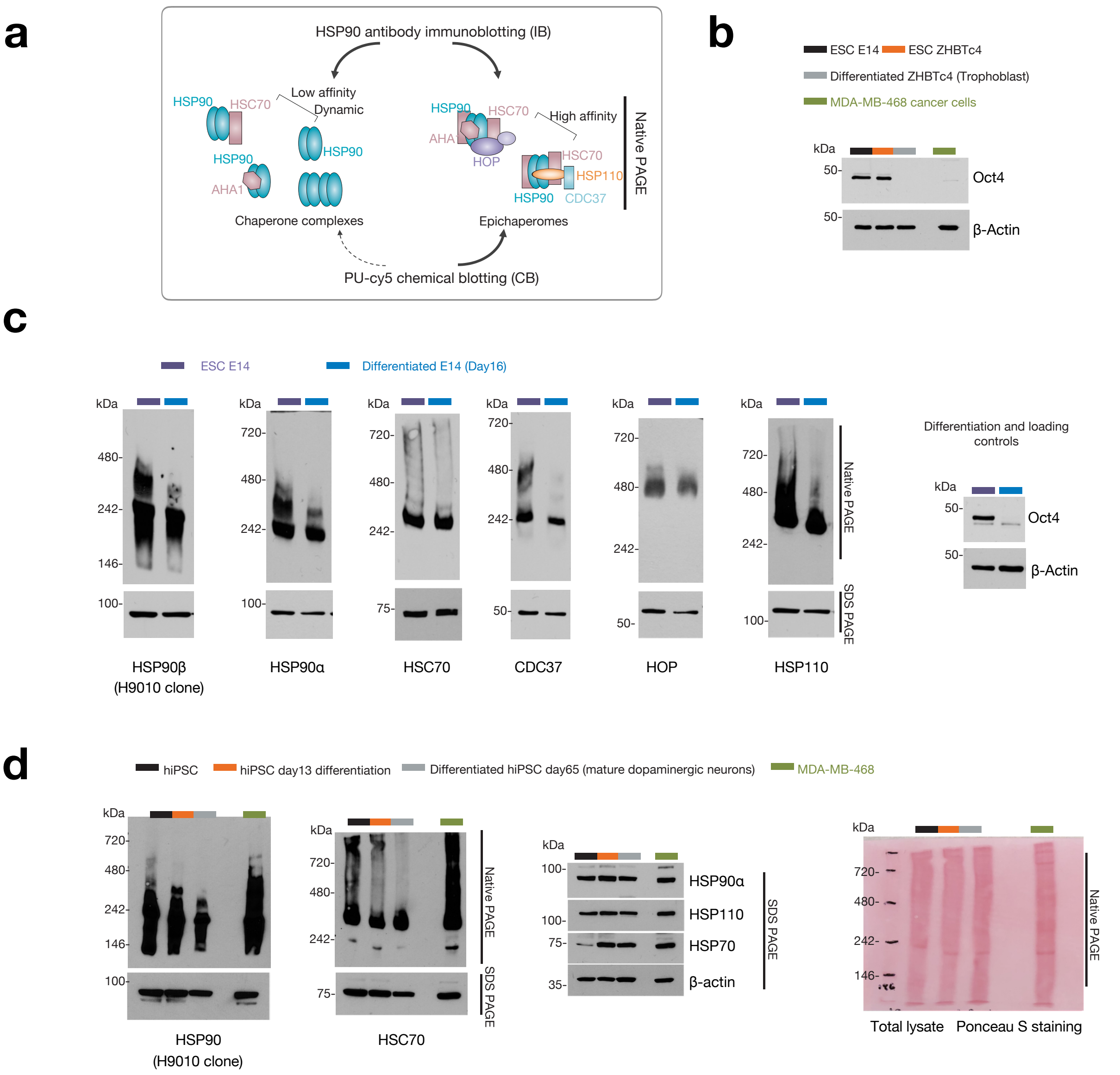

Supplementary Figure 1. Epichaperome analysis in pluripotent stem cells and differentiated cells reveals disassembly during differentiation. a Schematic illustrating the biochemical and functional distinctions between epichaperomes and traditional chaperones. The schematic also outlines key principles for the use of antibodies and PU-probes in epichaperome analysis. **b** Western blot analysis of Oct4 protein levels, serving as an indicator of pluripotency or differentiation for cells analyzed in Fig. 1. **c,d** Detection of epichaperome components (chaperones and co-chaperones) through SDS-PAGE (bottom, total protein levels) and native-PAGE (top), followed by immunoblotting in the indicated Pluripotent Stem Cells (PSCs) and differentiated cells. Differentiation and loading controls are also provided (right). Refer to Fig. 1 and Supplementary Fig. 2 for additional context. Gel images are representative of three independent experiments. Source data are provided as a Source data file.

Supplementary Figure 2

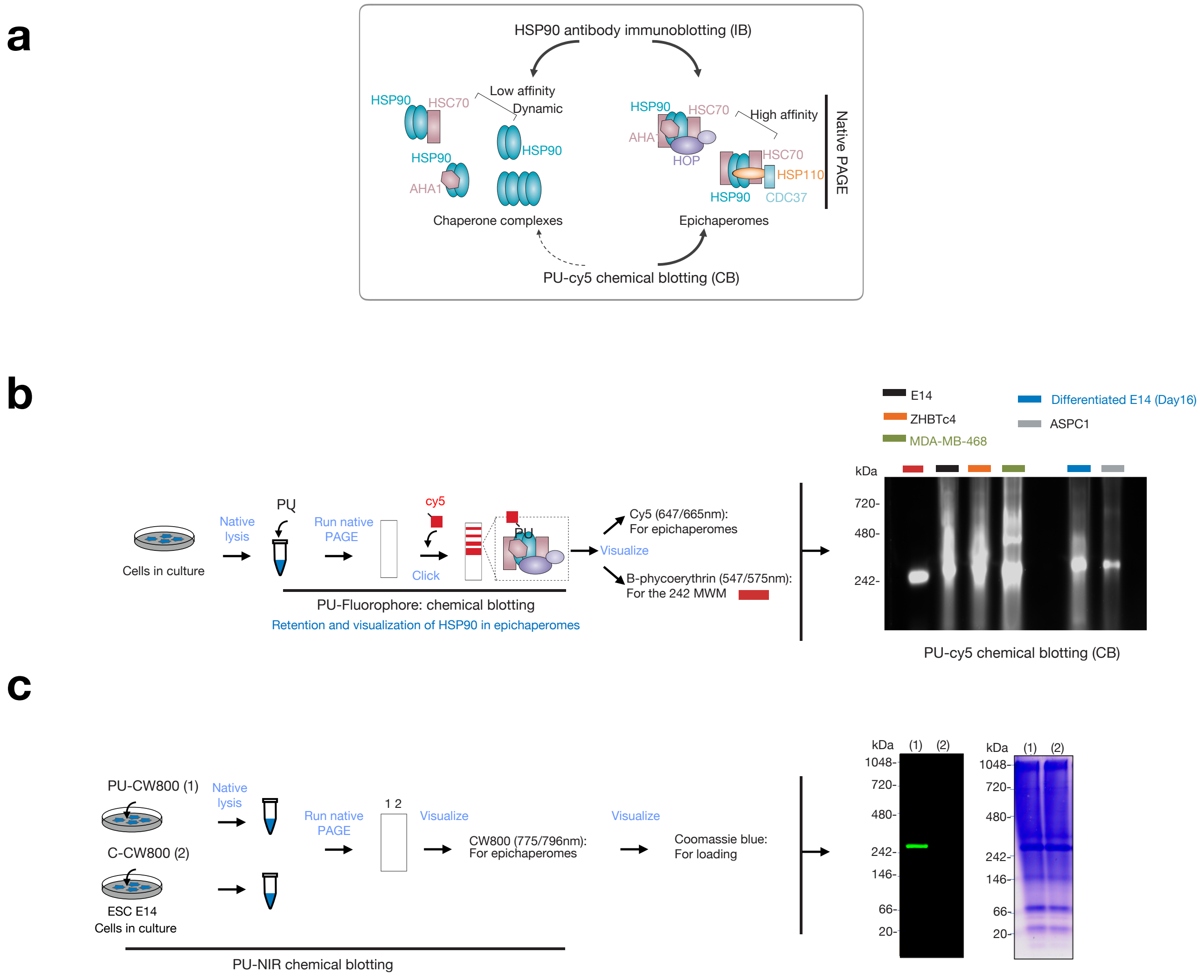

Supplementary Figure 2. Chemical blotting of epichaperomes in PSCs and cancer cells differentiated by epichaperome content. a Principle of epichaperome detection and preference for chemical probes and available antibodies. **b** Visualization of HSP90 in epichaperomes in cell homogenates using the PU-TCO probe clicked to Cy5. Schematic of the detection protocol (left) and representative gel (right, n = 3 independent experiments) showing epichaperome detection in cells with high epichaperome levels (E14 and ZHBTc4 ESCs and MDA-MB-468 cancer cells) and low epichaperome levels (ESCs differentiated to trophoblasts and ASPC1 cancer cells). See Fig. 1, Supplementary Fig. 1 and ref. Rodina et al. Nature 2016, these cells contain comparable levels of epichaperome components. **c** Visualization of the epichaperome core (~300 kDa) using PU-NIR (near infrared) chemical blotting. Schematic of the detection protocol (left) and representative gel (right, n = 3 independent experiments) are shown. PU-CW800, PU-H71 attached to a NIR dye, CW800; C-CW800, control probe. Coomassie blue staining, control to ensure consistent protein loading in both PU-CW800-treated and control-treated samples. Source data are provided as a Source Data file.

Supplementary Figure 3

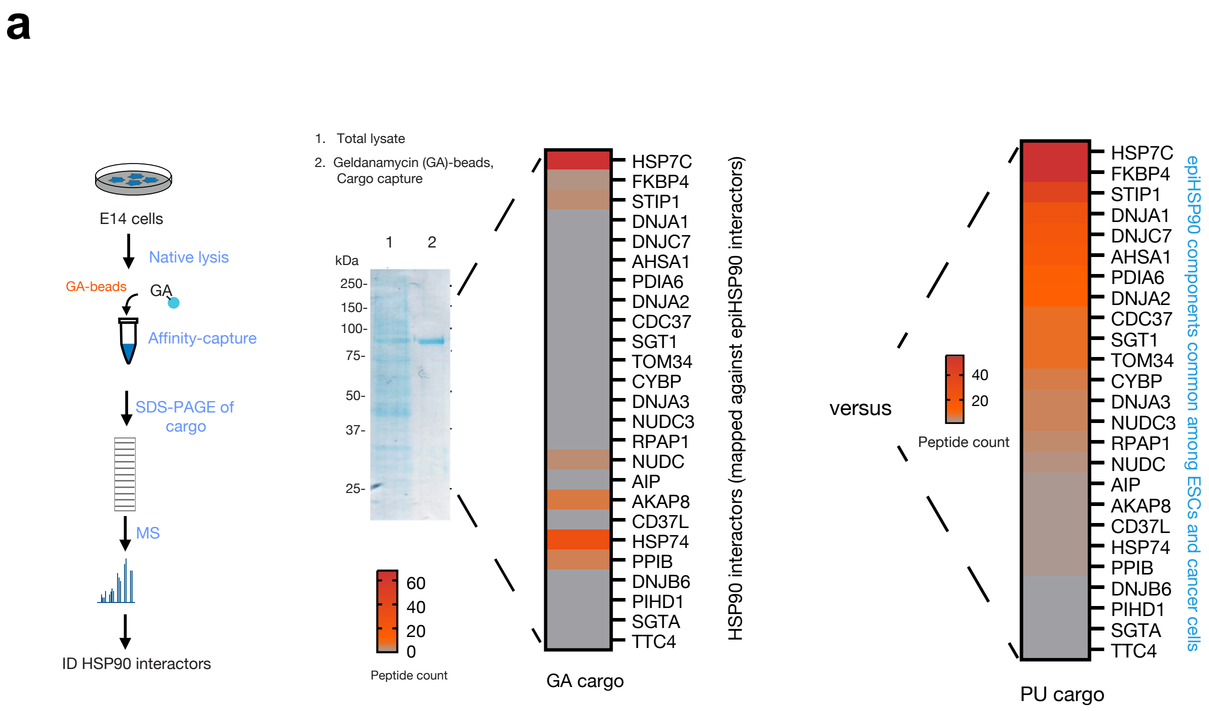

Supplementary Figure 3. Geldanamycin (GA) favors predominantly un-complexed HSP90. a Schematic of the capture experiment (left). Coomassie blue stained gel of the GA-bead cargo shows few interactors other than the predominant HSP90 band (middle). Heat map showing HSP90 interactor chaperones and co-chaperones isolated in the GA-cargo, identified by MS analysis. Interactors were mapped against the epichaperome component list identified in Rodina et al., Nature 2016. The PU-cargo is shown for comparison. Representative data of two independent experiments. Size bar, peptide count. Source data are provided in Supplementary Data 1.

Supplementary Figure 4

a

```
sp|P08238|HS90B_HUMAN      -----MPEEVHHGEEEVETFAFQAEIAQLMSLIINTFYSNKEIFLRELISNASDALDKIR 55
sp|P07900|HS90A_HUMAN      MPEETQTQDQPMEEEEVETFAFQAEIAQLMSLIINTFYSNKEIFLRELISNSSDALDKIR 60
                                  ::    **************************************:********

sp|P08238|HS90B_HUMAN      YESLTDPSKLDSGKELKIDIIPNPQERTLTLVDTGIGMTKADLINNLGTIAKSGTKAFME 115
sp|P07900|HS90A_HUMAN      YESLTDPSKLDSGKELHINLIPNKQDRTLTIVDTGIGMTKADLINNLGTIAKSGTKAFME 120
                           ****************:*::*** *:****:*****************************

sp|P08238|HS90B_HUMAN      ALQAGADISMIGQFGVGFYSAYLVAEKVVVITKHNDDEQYAWESSAGGSFTVRADHGEPI 175
sp|P07900|HS90A_HUMAN      ALQAGADISMIGQFGVGFYSAYLVAEKVTVITKHNDDEQYAWESSAGGSFTVRTDTGEPM 180
                           ****************************.************************:* ***:
                                                                                 226
sp|P08238|HS90B_HUMAN      GRGTKVILHLKEDQTEYLEERRVKEVVKKHSQFIGYPITLYLEKEREKEISDDEAEEEKG 235
sp|P07900|HS90A_HUMAN      GRGTKVILHLKEDQTEYLEERRIKEIVKKHSQFIGYPITLFVEKERDKEVSDDEAEEKED 240
                           **********************:**:**************::****:**:*******::.
                                                       255
sp|P08238|HS90B_HUMAN      ---EKEEEDKDDEEKPKIEDVGSDEEDDSGKDKKKKTKKIKEKYIDQEELNKTKPIWTRN 292
sp|P07900|HS90A_HUMAN      KEEEKEKEEKESEDKPEIEDVGSDEEEEKKDGDKKKKKKIKEKYIDQEELNKTKPIWTRN 300
                              ***:*:*:.*:**:*********::. ...***.***********************

sp|P08238|HS90B_HUMAN      PDDITQEEYGEFYKSLTNDWEDHLAVKHFSVEGQLEFRALLFIPRRAPFDLFENKKKKNN 352
sp|P07900|HS90A_HUMAN      PDDITNEEYGEFYKSLTNDWEDHLAVKHFSVEGQLEFRALLFVPRRAPFDLFENRKKKNN 360
                           *****:************************************:***********:*****

sp|P08238|HS90B_HUMAN      IKLYVRRVFIMDSCDELIPEYLNFIRGVVDSEDLPLNISREMLQQSKILKVIRKNIVKKC 412
sp|P07900|HS90A_HUMAN      IKLYVRRVFIMDNCEELIPEYLNFIRGVVDSEDLPLNISREMLQQSKILKVIRKNLVKKC 420
                           ************.*:****************************************:****

sp|P08238|HS90B_HUMAN      LELFSELAEDKENYKKFYEAFSKNLKLGIHEDSTNRRRLSELLRYHTSQSGDEMTSLSEY 472
sp|P07900|HS90A_HUMAN      LELFTELAEDKENYKKFYEQFSKNIKLGIHEDSQNRKKLSELLRYYTSASGDEMVSLKDY 480
                           ****:************** ****:******** **::*******:** *****.**.:*

sp|P08238|HS90B_HUMAN      VSRMKETQKSIYYITGESKEQVANSAFVERVRKRGFEVVYMTEPIDEYCVQQLKEFDGKS 532
sp|P07900|HS90A_HUMAN      CTRMKENQKHIYYITGETKDQVANSAFVERLRKHGLEVIYMIEPIDEYCVQQLKEFEGKT 540
                            :****.** *******:*:**********:**:*:**:** **************:**:

sp|P08238|HS90B_HUMAN      LVSVTKEGLELPEDEEEKKKMEESKAKFENLCKLMKEILDKKVEKVTISNRLVSSPCCIV 592
sp|P07900|HS90A_HUMAN      LVSVTKEGLELPEDEEEKKKQEEKKTKFENLCKIMKDILEKKVEKVVVSNRLVTSPCCIV 600
                           ******************** **.*:*******:**:**:******.:*****:******

sp|P08238|HS90B_HUMAN      TSTYGWTANMERIMKAQALRDNSTMGYMMAKKHLEINPDHPIVETLRQKAEADKNDKAVK 652
sp|P07900|HS90A_HUMAN      TSTYGWTANMERIMKAQALRDNSTMGYMAAKKHLEINPDHSIIETLRQKAEADKNDKSVK 660
                           **************************** *********** *:**************:**

sp|P08238|HS90B_HUMAN      DLVVLLFETALLSSGFSLEDPQTHSNRIYRMIKLGLGIDEDEVAAEEPNAAVPDEIPPLE 712
sp|P07900|HS90A_HUMAN      DLVILLYETALLSSGFSLEDPQTHANRIYRMIKLGLGIDEDDPTADDTSAAVTEEMPPLE 720
                           ***:**:*****************:****************: :*:: .*** :*:****

sp|P08238|HS90B_HUMAN      GDEDASRMEEVD 724
sp|P07900|HS90A_HUMAN      GDDDTSRMEEVD 732
                           **:*:*******
```

b

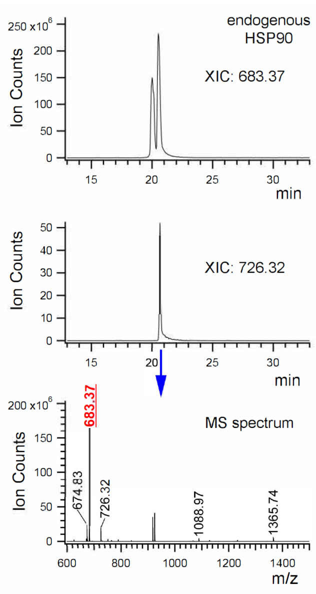

c

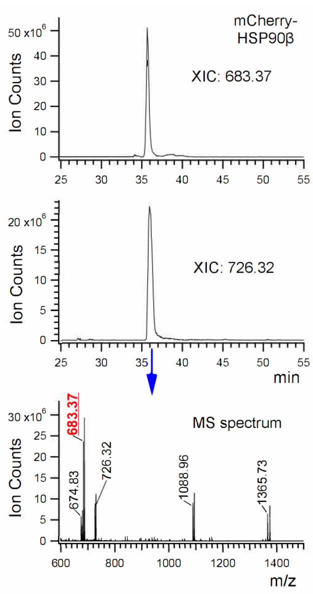

Supplementary Figure 4. HSP90 sequence alignment and isoform assignment. a The CLUSTAL multiple sequence alignment tool was employed to illustrate the similarities and distinctions between the two human HSP90 isoforms, HSP90α and HSP90β. The peptide sequence used to differentiate the isoforms in MS is highlighted in yellow, where a single amino acid distinguishes the isoforms (Ile in HSP90α and Leu in HSP90β). **b** Extracted Ion Chromatogram (XIC) of an isobaric peptide pair with m/z 683.37 from an endogenous HSP90 protein digest (top panel, b) reveals peptides from both HSP90α and HSP90β isoforms (two peaks). **c** XIC of the peptide from an mCherry-HSP90β protein digest (top panel, c) only contains the peptide from HSP90β isoform (one peak). Co-eluting peptides of the HSP90β isoform peptide (bottom panel, c) help distinguish the isobaric peptides and assign the second elution peak as the peptide from the HSP90β isoform (bottom panel, b). As expected, elution windows of the co-eluting peptide with m/z 726.32 align well with the isobaric peptide from HSP90β (middle panels).

Supplementary Figure 5

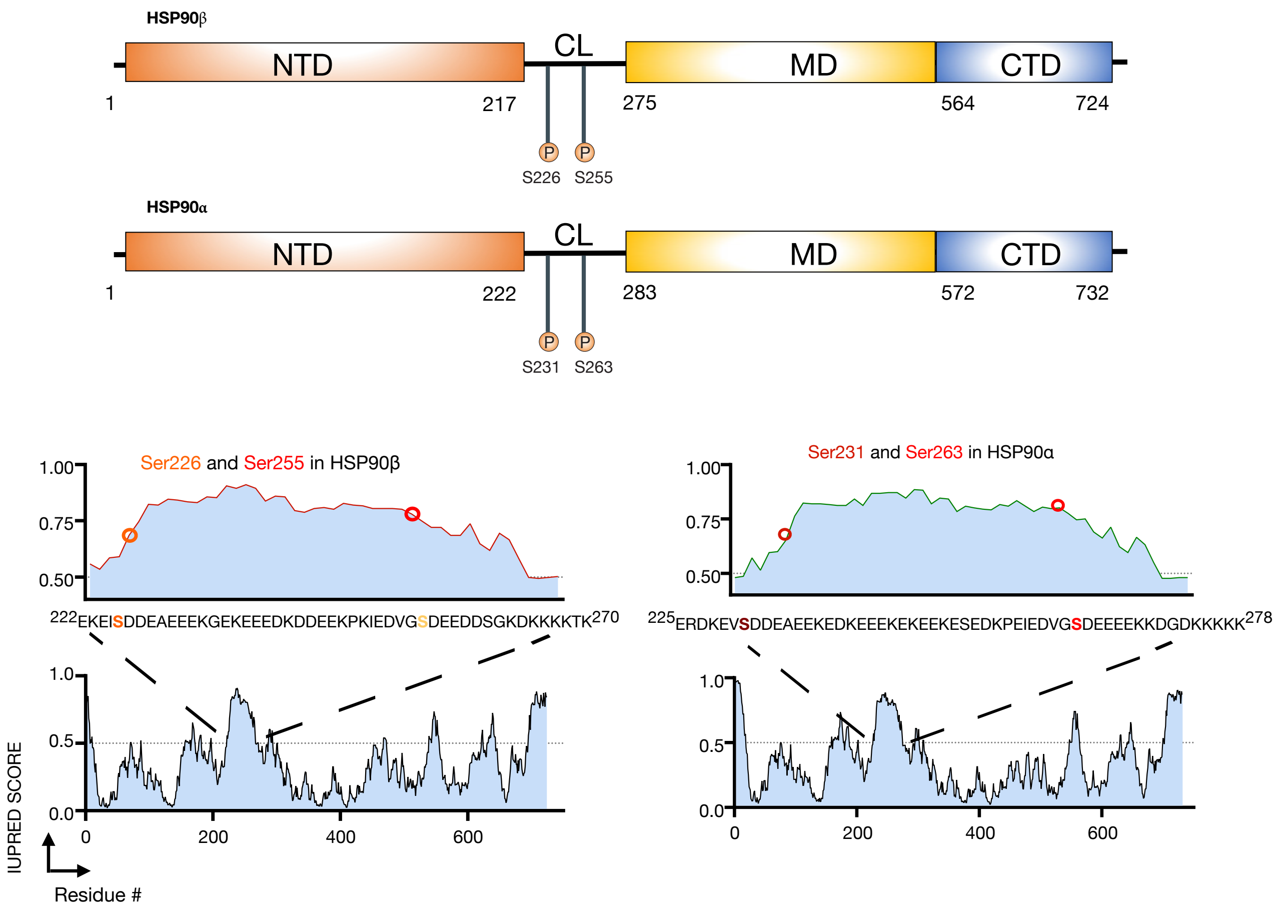

Supplementary Figure 5. Key residues that supports HSP90 incorporation into epichaperomes are located within an intrinsically disordered region (IDR) of HSP90. The IUPred tool predicted disorder in HSP90α (P07900, right) and HSP90β (P08238, left), respectively. The disorder prediction is given as a score ranging from 0 to 1, where higher scores indicate a higher likelihood of disorder. The charged linker (CL) and the location of the two key serine residues are also shown (top inset). NTD, N-terminal domain; MD, middle domain and CTD, C-terminal domain. Source data are provided as a Source Data file.

Supplementary Figure 6

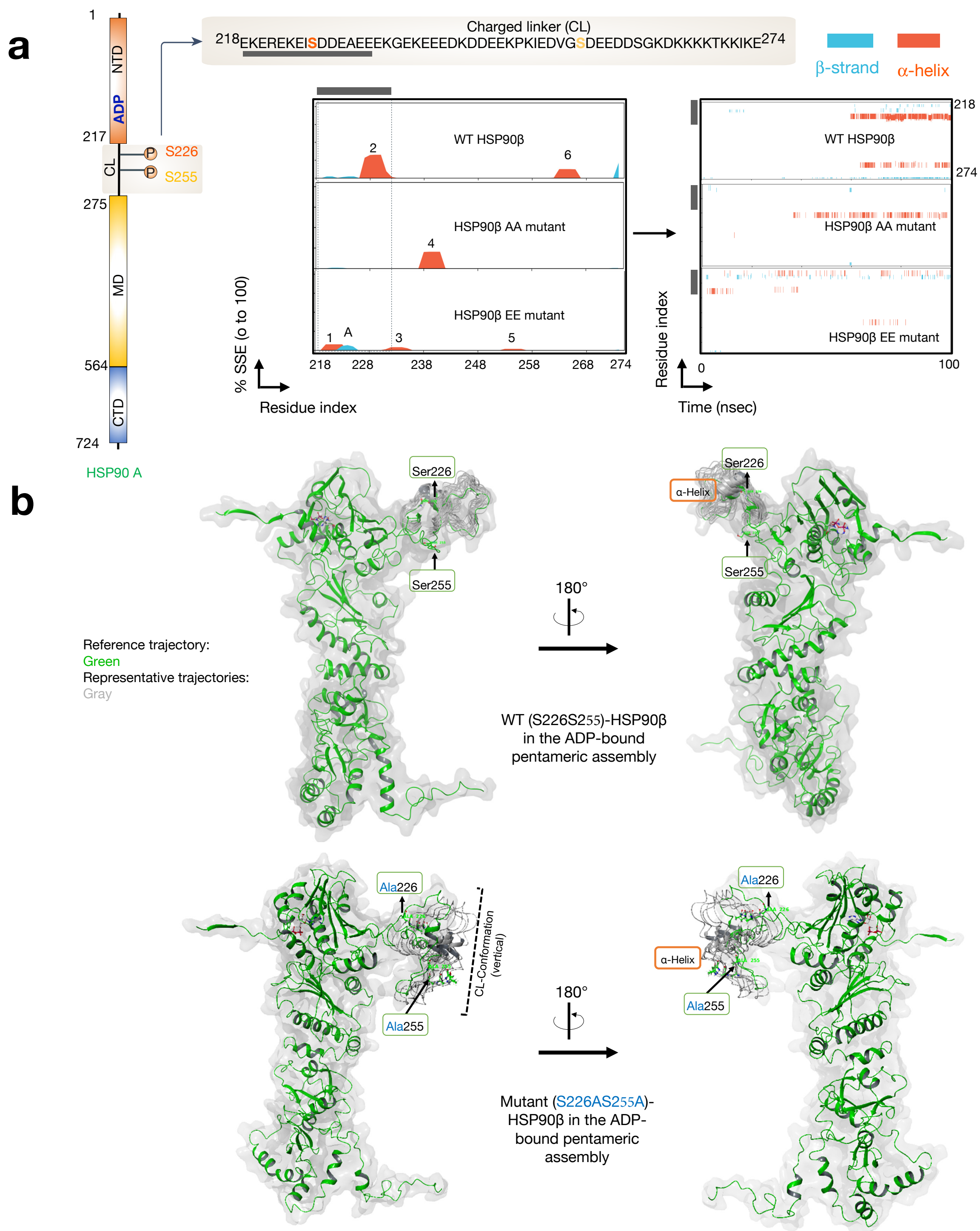

Supplementary Figure 6. The conformational shift induced in the HSP90 charged linker upon phosphorylation is sensitive to the identity of the ligand a Protein secondary structure elements (SSE) like alpha-helices and beta-strands of the charged linker of protomer A of ADP-bound HSP90-containing assemblies monitored throughout the MD simulation. Pentameric assemblies with WT (HSP90 S226/S255), phosphomimetic (HSP90 S226E/S255E) and non-phosphorylatable (HSP90 S226A/S255A) mutants were analyzed. The plot on the left reports SSE distribution by residue index throughout the charged linker and the plot on the right monitors each residue and its SSE assignment over time. Schematic illustrating the primary structure of the full-length HSP90 with color-coded domains is also shown: NTD, N-terminal domain; MD, middle domain and CTD, C-terminal domain. The charged linker (CL) and the location of the two key serine residues are also shown (top inset). The gray bar indicates the CL segment encompassing residues 218 to 232. **b** Ribbon representation of ADP-bound HSP90 protomer A in assemblies containing the WT (HSP90 S226/S255) or the non-phosphorylatable (HSP90 S226A/S255A) mutants is shown. Despite a higher content of structured elements, such as alpha-helices, the up conformational flip of the charged linker is not attainable. Therefore, the strategic formation of beta-sheet A, rather than the overall structured nature of the charged linker, is crucial for inducing the up conformation. Trajectories, reference and representative, of n = 1,000 simulations are shown. Each condition was simulated three times with similar results.

Supplementary Figure 7

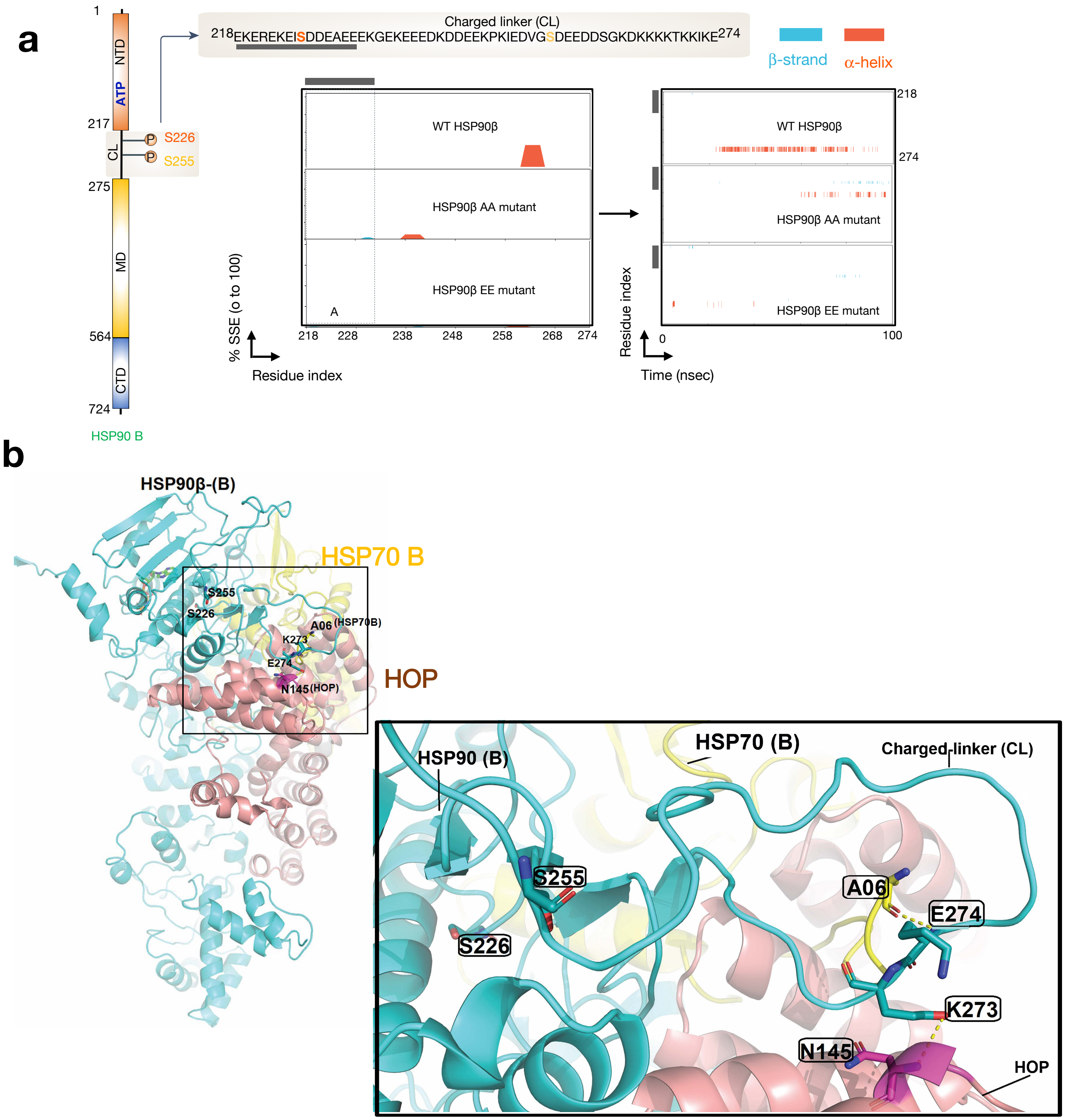

Supplementary Figure 7. Structural analysis of the charged linker in HSP90 protomer B within ATP-bound assemblies. a Protein secondary structure elements (SSE) like alpha-helices and beta-strands of the charged linker of protomer B of ATP-bound HSP90-containing assemblies monitored throughout the MD simulation. Pentameric assemblies with WT (HSP90 S226/S255), phosphomimetic (HSP90 S226E/S255E) and non-phosphorylatable (HSP90 S226A/S255A) mutants were analyzed. The plot on the left reports SSE distribution by residue index throughout the charged linker and the plot on the right monitors each residue and its SSE assignment over time. Schematic illustrating the primary structure of the full-length HSP90 with color-coded domains is also shown: NTD, N-terminal domain; MD, middle domain and CTD, C-terminal domain. The charged linker (CL) and the location of the two key serine residues are also shown (top inset). The gray bar indicates the CL segment encompassing residues 218 to 232. **b** Cartoon representation of ATP-bound HSP90 protomer B in assemblies containing the EE mutant is shown. In the assembly, the presence of HSP70(B) and HOP results in stabilizing intermolecular hydrogen-bond interactions with the charged linker of HSP90(B). These interactions effectively lock the linker into a specific conformation, thereby limiting its potential for SSE formation and conformational rearrangement compared to protomer A. Each condition was simulated three times with similar results.

Supplementary Figure 8

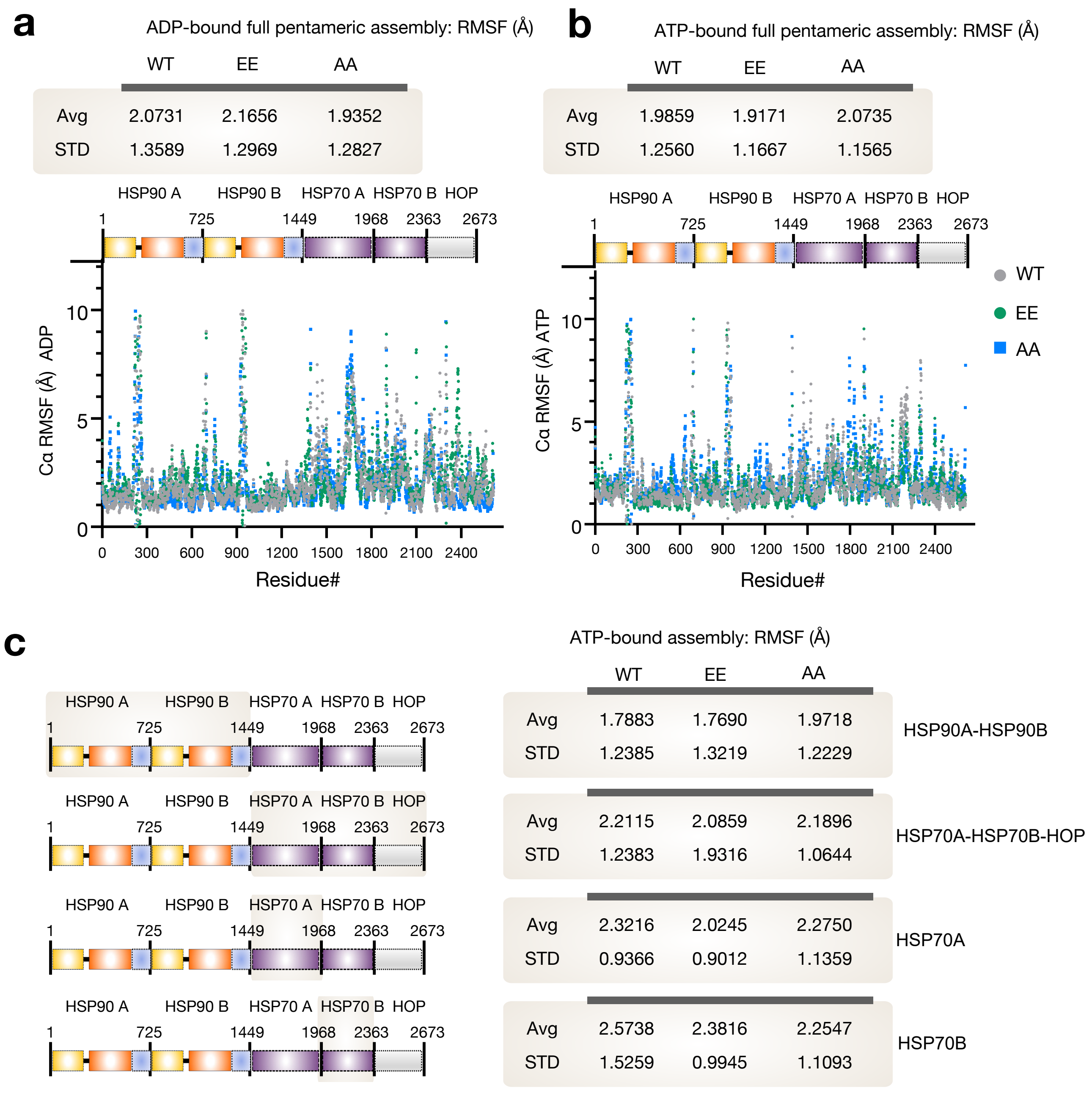

Supplementary Fig. 8. Flexibility analysis of assembly components in ADP- and ATP-bound HSP90 pentameric complexes. a,b The plot depicts the Root Mean Square Fluctuation (RMSF) values for each residue within the ADP-bound (**a**) and ATP-bound (**b**) pentameric assemblies across different conditions. The pentameric assemblies were prepared in the following combinations: 2xHSP90(Ser226Ser255)-2xHSP70-HOP, 2xHSP90(Glu226Glu255)-2xHSP70-HOP-, 2xHSP90(Ala226Ala255)-2xHSP70-HOP, each bound to either ATP or ADP. Each point along the x-axis corresponds to a specific residue in the protein sequence of HSP90A, HSP90B, HSP70A, HSP70B and HOP. The y-axis represents the RMSF value in angstroms (Å), indicating the average flexibility of each residue. Higher RMSF values suggest greater flexibility, while lower values indicate rigidity. **c** The RMSF values were also calculated as the average ± standard deviation (STD) of all residues for each protein component or over several components, allowing us to pinpoint regions (or components) within the assemblies that exhibit higher flexibility or rigidity. Each condition was simulated three times, for 100 ns each, yielding similar results. See also Supplementary Data 5 for source data.

Supplementary Fig. 9

a

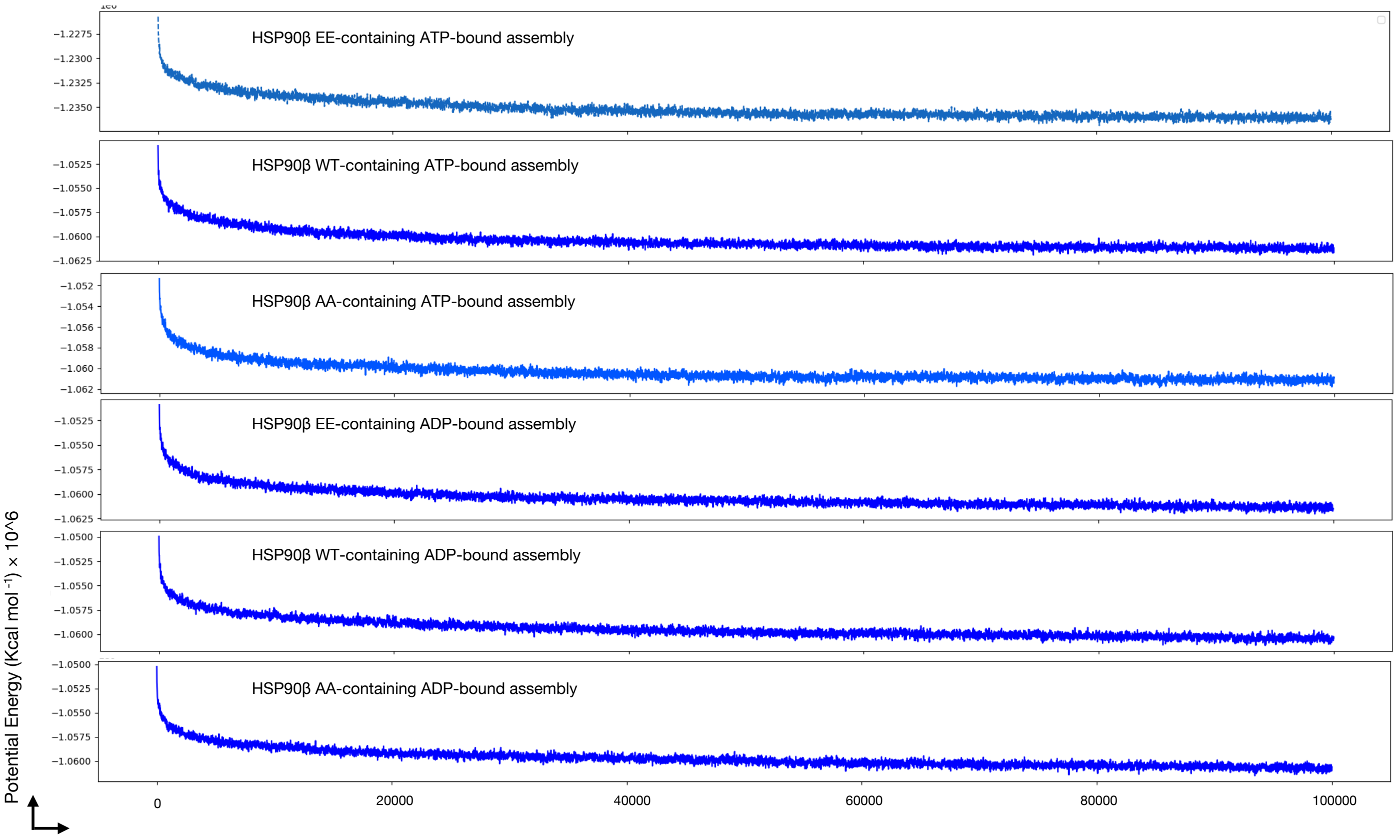

b

ATP-bound assembly	Potential Energy (Kcal.mol^{-1})	Total Energy (Kcal.mol^{-1})
HSP90β EE	-1235175.2	-1034356.4
HSP90β WT	-1060447.3	-886519.8
HSP90β AA	-1060393.3	-886471.2

ADP-bound assembly	Potential Energy (Kcal.mol^{-1})	Total Energy (Kcal.mol^{-1})
HSP90β EE	-1060424.4	-886518.7
HSP90β WT	-1059442.9	-885586.7
HSP90β AA	-1059722.4	-885780.3

Supplementary Figure 9. Potential and total energies of ATP- and ADP-bound assemblies. a Graphs showing the potential energy versus production simulation time (ps) for ATP-bound and ADP-bound assemblies. Each graph represents the stability of the assemblies containing HSP90β EE, WT, or AA mutants. **b** Table summarizing the potential and total energies for ATP-bound and ADP-bound assemblies.

Supplementary Fig. 10

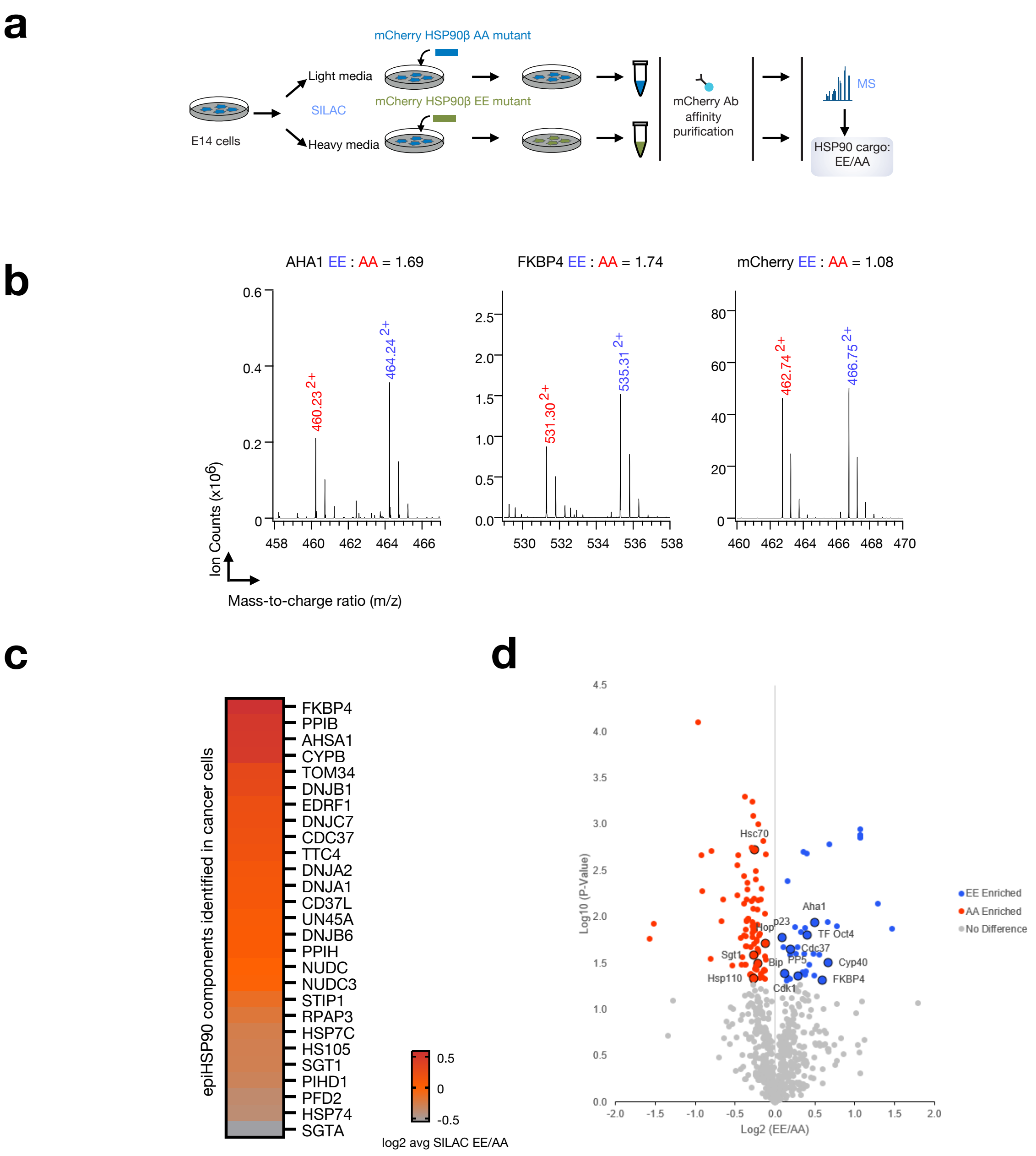

Supplementary Figure 10. Immunopurification reveals increased presence of epichaperome-specific co-chaperones in phosphomimetic HSP90 complexes compared to non-phosphorylatable complexes. a Experiment outline and outcomes. **b** Representative spectra (n = 3 independent experiment) of proteins co-purified with the phosphomimetic $HSP90^{S226E,S255E}$ (EE) and non-phosphorylatable $HSP90^{S226A,S255A}$ (AA) mutants. **c** Heatmap showing the identity of chaperone and co-chaperones identified as epichaperome components in cancer cells (as per Rodina et al. Nature 2016) and enriched in the affinity purified $HSP90^{S226E,S255E}$ mutant. Scale bar, log2 average SILAC values EE/AA (n = 3). **d** Volcano plot of the entire interactome identified as in panel a. The statistically significant changes are highlighted as indicated. Source data are provided as a Source Data file and as Supplementary Data 7.

Supplementary Figure 11

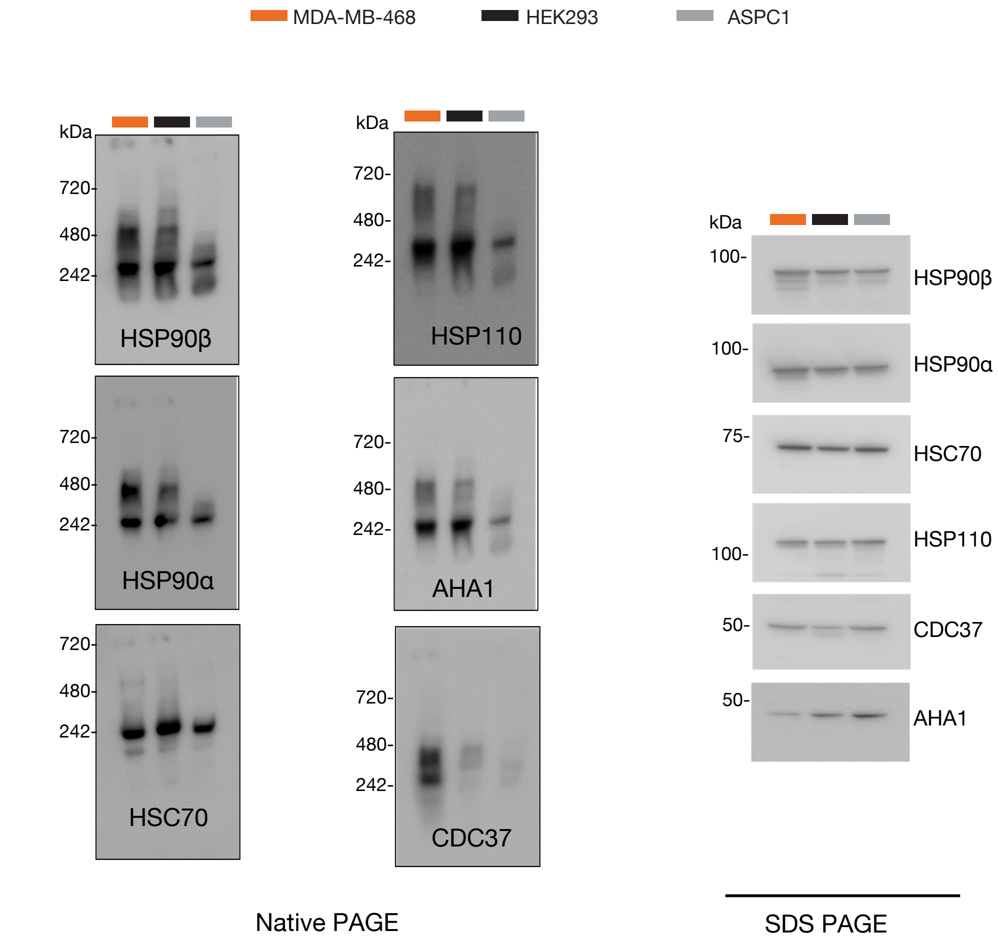

Supplementary Figure 11. HEK293 cells express epichaperomes. a Detection of epichaperome components (chaperones and co-chaperones) through SDS-PAGE (bottom, total protein levels) and native-PAGE (top), followed by immunoblotting in the indicated cells. Gel images are representative of three independent experiments. Source data are provided as a Source data file.

Supplementary Figure 12

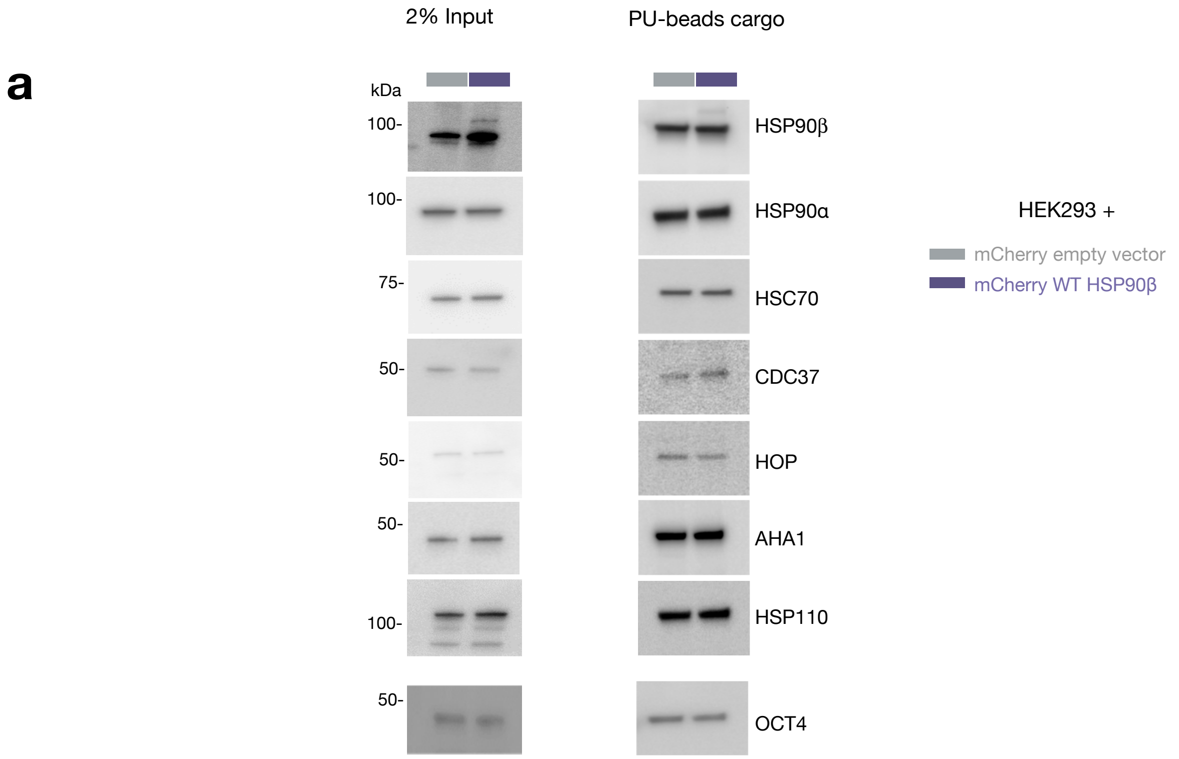

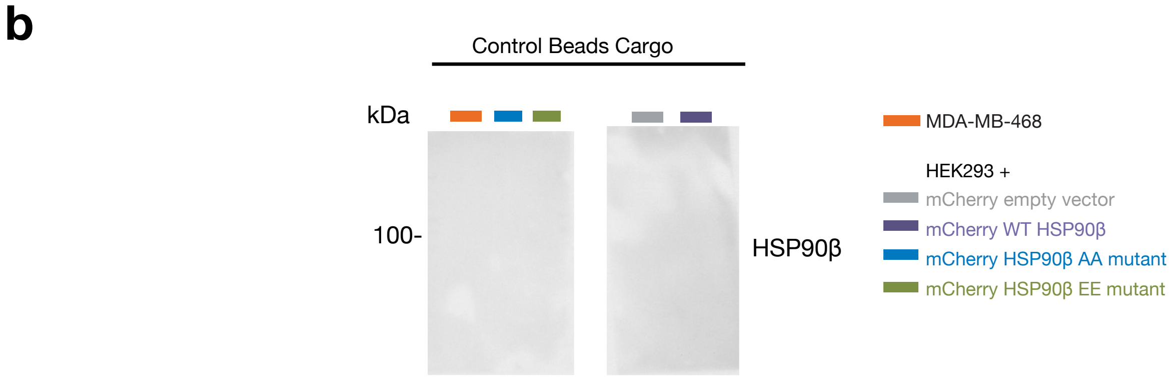

Supplementary Figure 12. Overexpression of the WT HSP90 in HEK293 cells has only a minor impact on endogenous epichaperomes. a Detection and quantification of epichaperome components through PU-beads capture as indicated in Fig. 6a schematic. See also Fig. 6c for epichaperome detection via native PAGE followed by immunoblotting. Protein amount loaded for Input represents 2% of the protein amount incubated with the beads. **b** Capture with control beads is shown to confirm probe specificity. Gel images are representative of three independent experiments. Source data are provided as Source data file.

Supplementary Figure 13

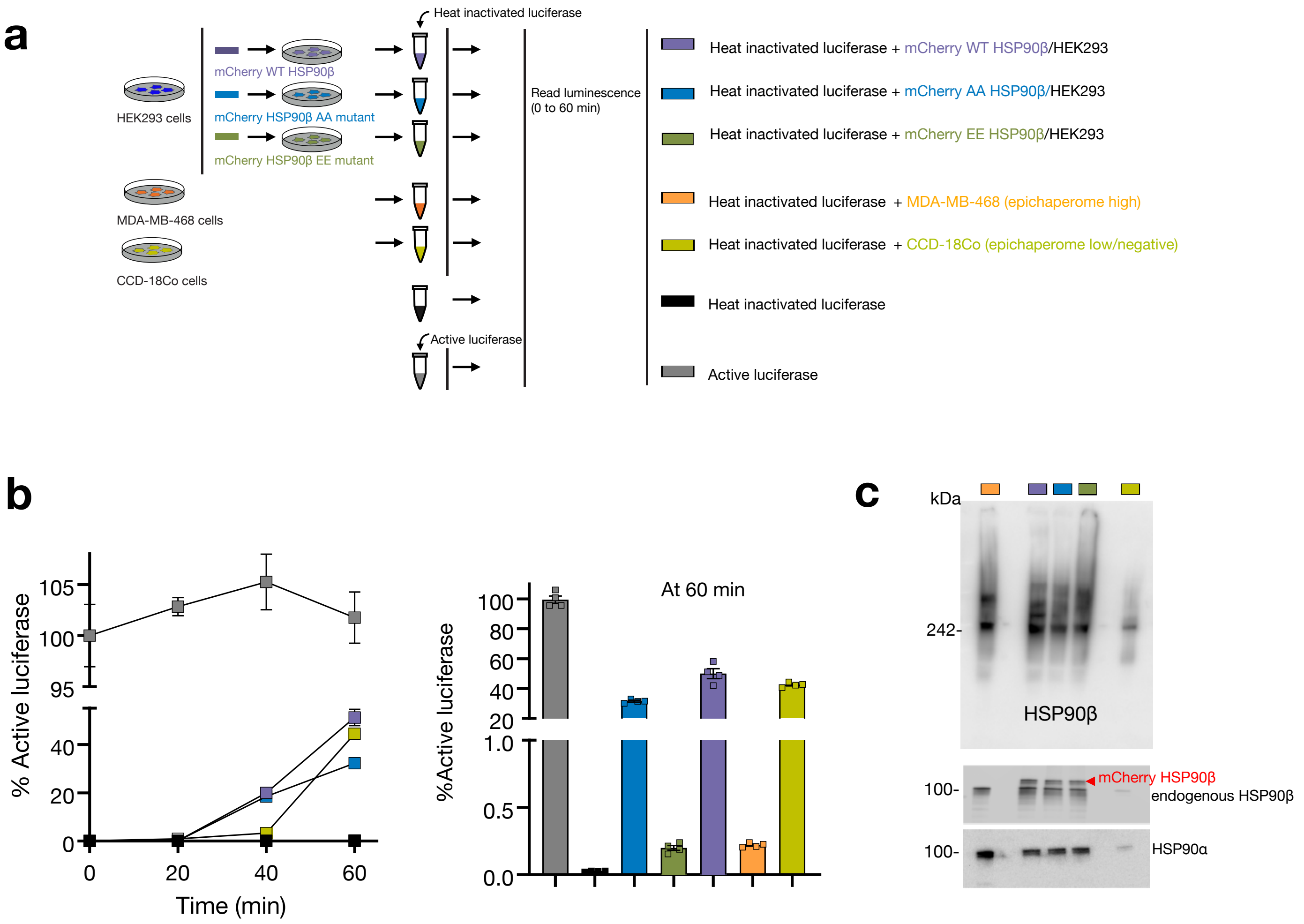

Supplementary Figure 13. Phosphorylation of HSP90 at key serine residues impairs its ability to refold denatured proteins. a Schematic. Refolding of heat denatured luciferase was investigated using denatured luciferase as a substrate to assess the refolding capabilities of different HSP90 mutants present in HEK293 cell lysates. We prepared cell extracts from HEK293 cells transfected with cherry-HSP90β constructs, specifically the WT, AA (non-phosphorylatable), and EE (phosphomimetic) mutants. MDA-MB-468 cells were used as a control for a cell line with endogenously high epichaperome content, and CCD-18Co cells were used as a control for a cell line with low/negative epichaperome content. Denatured luciferase was mixed with equal amounts of these lysates to determine whether the distinct HSP90 species in each lysate could facilitate the refolding of luciferase. **b** The graphs represents luciferase activity readings taken 0 to 60 minutes (left) and at 60 min (right) after mixing denatured luciferase with the lysates. Active luciferase was used as a 100% activity control. Data represent mean ± s.e.m., with n = 4 biological replicates. **c** Epichaperome levels (top gel) and HSP90 levels (bottom gels) of the lysates analyzed in the luciferase refolding assay as shown in panels a and b. Source data are provided as Source data file.

Supplementary Figure 14

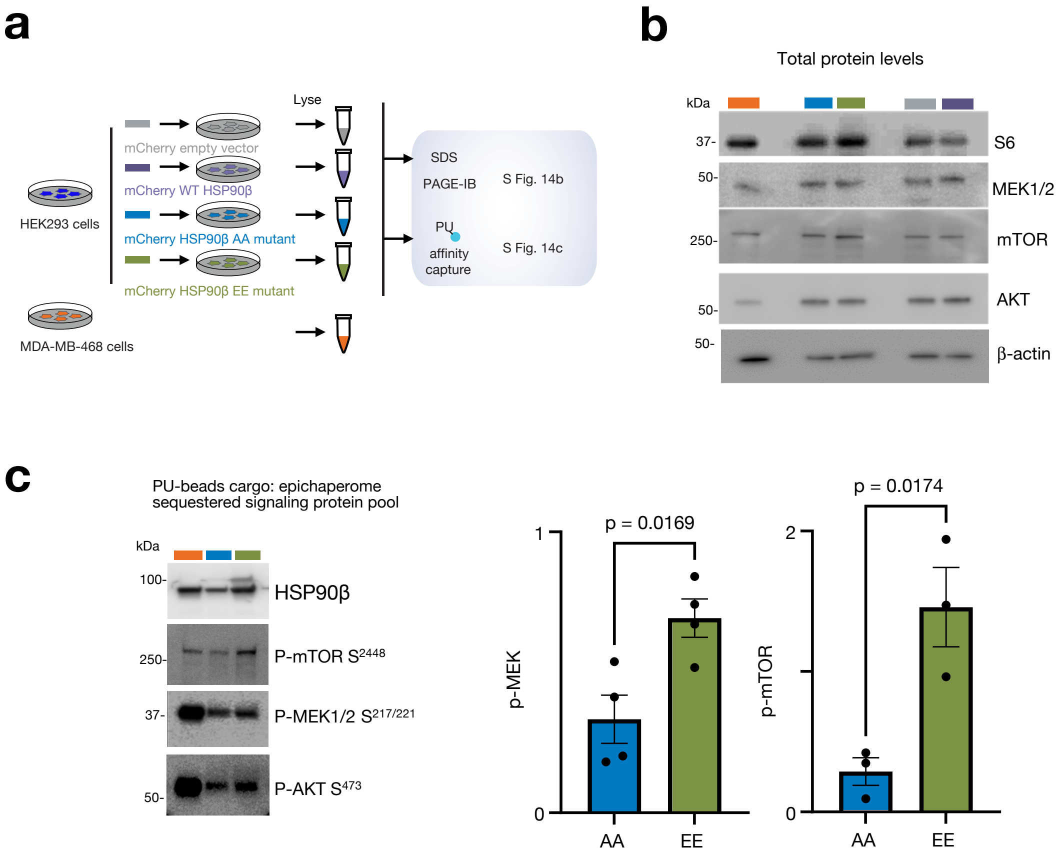

Supplementary Figure 14. The effect of HSP90 mutants on signaling proteins. a Overview of the experimental design and expected outcomes (panels b,c). See also Fig. 8. **b** Detection of total protein levels in cells transfected with the indicated HSP90 mutants or vector control, as observed through western blot analysis. The analysis includes proteins involved in transducing signaling events related to cell proliferation, survival, and protein synthesis control. Gels represent three independent experiments. **c** Active signaling proteins sequestered into the epichaperome platforms, identified through PU-beads capture. HSP90β serves as the capture control. MDA-MB-468 serves as an internal standard and quantification control. Phosphomimetic HSP90β$S^{226E,S255E}$ (EE); nonphosphorylatable HSP90^{S226A,S255A} (AA). Data are presented as mean ± s.e.m., p-MEK n = 4, p-mTOR, n = 3, using unpaired two-tailed t-test for statistical analysis. Source data are provided as Source data file.

Supplementary Figure 15

a

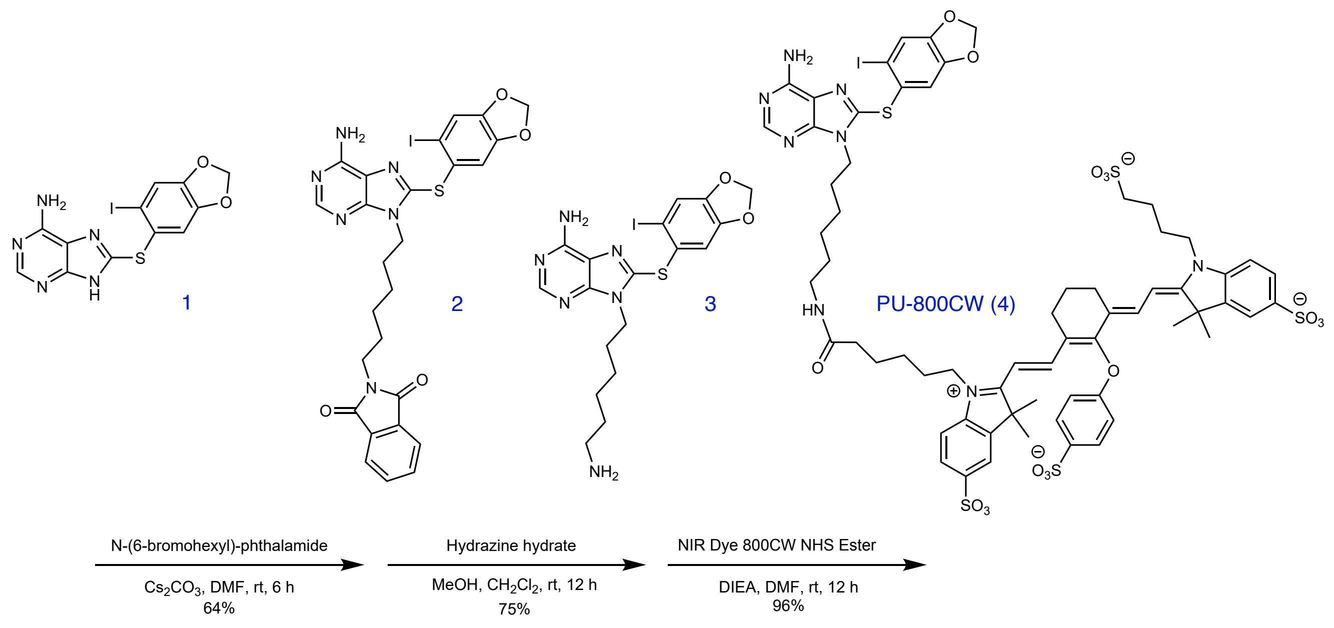

b

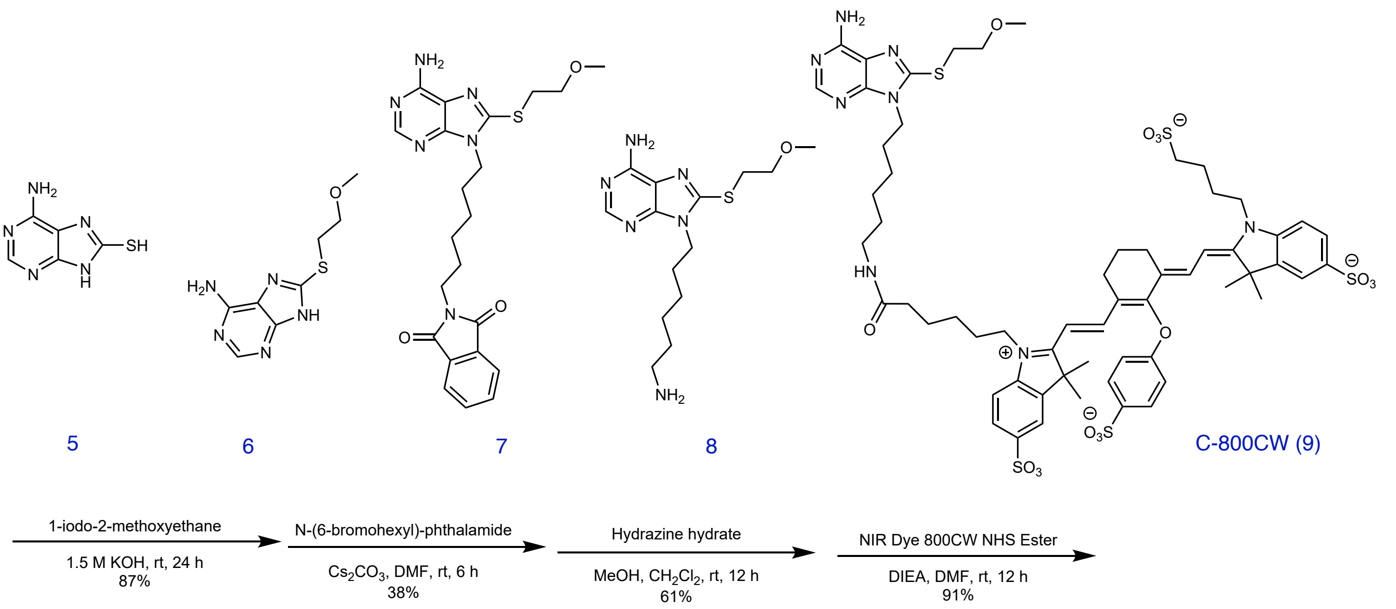

Supplementary Figure 15. Synthetic scheme for the preparation of PU-800CW and NC-800CW. a Synthesis of PU-800CW (4) commenced with the reaction of compound 1 with N-(6-bromohexyl)-phthalamide, followed by treating the resulting compound 2 with hydrazine hydrate to yield intermediate 3. Compound 3 was then coupled with near-infrared (NIR) Dye 800CW NHS Ester to obtain PU-800CW (4). **b** Synthesis of NC-800CW started from 5. Sequential reaction of 5 with 1-iodo-2-methoxyethane followed by alkylation with N-(6-bromohexyl)-phthalamide resulted in the intermediate 7. Treating 7 with hydrazine hydrate yielded compound 8, which was then coupled with NIR Dye 800CW NHS Ester in the presence of DIEA to yield C-800CW (9). See Supplementary Note 1 for detailed description of probe synthesis, purification, and characterization. Cs_2CO_3, Cesium Carbonate; DMF, Dimethyl Formamide; MeOH, Methanol; CH_2Cl_2; Dichloromethane; NIR Dye 800CW NHS Ester, Near-Infrared Dye 800CW N-Hydroxysuccinimide Ester; DIEA, N,N-Diisopropylethylamine; KOH, Potassium Hydroxide, rt, room temperature.

Supplementary Table 1. Sequence coverage for mCherry-HSP90Beta-WT construct showing 434x coverage.

>mCherry-HSP90B-WT coverage: 434x

AAAAAAAATCTAACCTCAAGTCAAGGCTTTTCTATGGAATAAGGAATGGACAGCAGGGGGCTGTTTCATATACTGATGACCTCTTTATAGCCAACCTTTGTTCATGGCAGCCA
GCATATGGGCATATGTTGCCAAACTCTAAACCAAATACTCATTCTGATGTTTTAAATGATTTGCCCTCCCATATGTCCTTCCGAGTGAGAGACACAAAAAATTCCAACACACTA
TTGCAATGAAAATAAATTTCCTTTATTAGCCAGAAGTCAGATGCTCAAGGGGCTTCATGATGTCCCCATAATTTTTGGCAGAGGGAAAAAGATTGAGATCTTCCCTAATCGACT
TCTTCCATGCGAGACGCATCCTCATCGCCCTCGAGAGGGGGGATCTCATCAGGAACTGCAGCATTGGGTTCCTCTGCTGCCACTTCATCTTCATCAATACCTAGACCTAGCT
TGATCATGCGATAGATGCGGTTGGAGTGGGTCTGGGGATCCTCAAGGGAAAAGCCAGAAGATAGCAGGGCGGTTTCAAACAGCAGCACCACCAGGTCCTTAACTGCCTTAT
CATTCTTGTCGGCCTCAGCCTTCTGCCGCAGCGTCTCCACAATGGGGTGGTCAGGGTTGATCTCCAGGTGCTTTTTGGCCATCATATAGCCCATGGTGGAGTTGTCCCGAA
GTGCCTGGGCTTTCATGATCCGCTCCATATTGGCTGTCCAGCCGTAGGTGCTGGTCACAATGCAGCAAGGTGAAGACACAAGTCTATTGGAGATTGTCACCTTCTCAACCTT
CTTATCTAAGATTTCTTTCATGAGCTTGCAGAGGTTCTCAAACTTTGCCTTGCTCTCTTCCATCTTCTTCTTCTCCTCCTCATCCTCAGGCAGCTCCAGACCCTCCTTGGTAAC
TGAGACCAGGCTCTTCCCATCAAATTCCTTGAGCTGCTGCACACAGTACTCGTCAATGGGCTCGGTCATATATACCACCTCGAAGCCCCGTTTCCGCACTCGCTCCACAAAA
GCTGAGTTGGCCACCTGCTCTTTGCTCTCACCAGTGATGTAATAGATGGACTTCTGTGTCTCCTTCATGCGAGAAACATACTCTGACAGAGATGTCATCTCATCTCCAGACTG
GGAGGTATGATAGCGCAGCAGCTCAGACAGGCGGCGGCGGTTAGTGGAGTCTTCGTGGATTCCAAGCTTGAGATTTTTAGAGAATGCCTCATAGAATTTCTTGTAATTCTCC
TTGTCTTCTGCCAGCTCAGAGAAGAGCTCAAGGCACTTCTTAACAATGTTTTTGCGAATGACTTTCAAGATTTTGCTCTGCTGGAGCATTTCTCGGGAGATGTTCAGGGGCAG
ATCCTCAGAGTCAACCACACCACGGATAAAATTGAGATACTCTGGTATCAACTCATCACAGCTGTCCATGATGAACACACGGCGGACATAGAGTTTGATGTTGTTCTTTTTCT
TCTTGTTCTCAAAAAGGTCAAAGGGAGCCCGACGAGGAATAAATAGCAATGCCCTGAATTCCAACTGACCTTCTACAGAAAAGTGCTTGACTGCCAAGTGGTCTTCCCAGTC
ATTAGTGAGGCTCTTGTAGAATTCTCCATACTCCTCTTGGGTGATGTCATCAGGGTTTCTGGTCCAAATAGGCTTGGTCTTGTTTAGTTCTTCCTGATCAATGTATTTCTCTTTG
ATCTTCTTAGTTTTCTTCTTCTTATCCTTACCGCTGTCATCCTCCTCATCTGAACCCACATCTTCAATCTTGGGCTTTTCTTCATCATCTTTATCTTCCTCTTCTTTCTCACCTTTC
TCTTCCTCTGCCTCATCATCACTAATTTCCTTCTCTCGTTCCTTCTCCAAATAAAGGGTGATGGGATAGCCTATGAACTGAGAATGCTTCTTCACTACTTCTTTGACCCGCCTC
TCTTCTAGGTACTCTGTCTGATCTTCTTTAAGATGGAGGATCACTTTGGTACCCCTGCCAATGGGCTCACCATGGTCAGCACGCACAGTGAAGGAACCTCCAGCAGAAGACT
CCCAAGCATACTGTTCATCATCGTTGTGCTTTGTGATCACAACCACTTTCTCTGCCACCAAGTAGGCAGAATAAAAGCCAACACCAAACTGCCCAATCATGGAGATGTCTGCA
CCAGCCTGAAGAGCCTCCATGAATGCTTTAGTACCAGACTTGGCAATGGTTCCCAAATTATTTATGAGATCAGCTTTGGTCATGCCAATGCCTGTGTCAACCAAAGTCAGGGT
ACGTTCCTGAGGGTTGGGGATGATGTCAATTTTCAGCTCTTTACCACTGTCCAACTTCGAAGGGTCTGTCAGGCTCTCATAGCGAATCTTGTCCAAGGCATCAGAAGCATTA
GAGATCAACTCCCGAAGGAAAATCTCCTTGTTGGAATAGAAGGTATTGATGATGAGGGACATGAGTTGGGCAATTTCTGCCTGAAAGGCAAAAGTCTCCACCTCCTCCTCTC
CATGGTGCACTTCCTCTGGCATCGGTCCGGACTTGTACAGCTCGTCCATGCCGCCGGTGGAGTGGCGGCCCTCGGCGCGTTCGTACTGTTCCACGATGGTGTAGTCCTCG
TTGTGGGAGGTGATGTCCAACTTGATGTTGACGTTGTAGGCGCCGGGCAGCTGCACGGGCTTCTTGGCCTTGTAGGTGGTCTTGACCTCAGCGTCGTAGTGGCCGCCGTC
CTTCAGCTTCAGCCTCTGCTTGATCTCGCCCTTCAGGGCGCCGTCCTCGGGGTACATCCGCTCGGAGGAGGCCTCCCAGCCCATGGTCTTCTTCTGCATTACGGGGCCGT
CGGAGGGGAAGTTGGTGCCGCGCAGCTTCACCTTGTAGATGAACTCGCCGTCCTGCAGGGAGGAGTCCTGGGTCACGGTCACCACGCCGCCGTCCTCGAAGTTCATCAC
GCGCTCCCACTTGAAGCCCTCGGGGAAGGACAGCTTCAAGTAGTCGGGGATGTCGGCGGGGTGCTTCACGTAGGCCTTGGAGCCGTACATGAACTGAGGGGACAGGATG
TCCCAGGCGAAGGGCAGGGGGCCACCCTTGGTCACCTTCAGCTTGGCGGTCTGGGTGCCCTCGTAGGGGCGGCCCTCGCCCTCGCCCTCGATCTCGAACTCGTGGCCGT
TCACGGAGCCCTCCATGTGCACCTTGAAGCGCATGAACTCCTTGATGATGGCCATGTTATCCTCCTCGCCCTTGCTCACCATGGTGGCGCTAGAATTCTTTGCCAAAATGAT
GAGACAGCACAATAACCAGCACGTTGCCCAGGAGCTGTAGGAAAAAGAAGAAGGCATGAACATGGTTAGCAGAGGCTCTAGAGCCGCCGGTCACACGCCAGAAGCCGAAC
CCCGCCCTGCCCCGTCCCCCCCGAAGGCAGCCGTCCCCCCGCGGACAGCCCCGAGGCTGGAGAGGGAGAAGGGGACGGCGGCGCGGCGACGCACGAAGGCCCTCCCC
GCCCATTTCCTTCCTGCCGGCGCCGCACCGCTTCGCCCCGCGCCCGCTAGAGGGGGTGCGGCGGCGCCTCCCAGATTTCGGCTCCGCACAGATTTGGGACAAAGGAAGT
CCCTGCGCCCTCTCGCACGATTACCATAAAAGGCAATGGCTGCGGCTCGCCGCGCCTCGACAGCCGCCGGCGCTCCGGGGGCCGCCGCGCCCCTCCCCCGAGCCCTCC
CCGGCCCGAGGCGGCCCCGCCCCGCCCGGCACCCCCACCTGCCGCCACCCCCCGCCCGGCACGGCGAGCCCCGCGCCACGCCCCGTACGGAGCCCCGCACCCGAAG
CCGGGCCGTGCTCAGCAACTCGGGGAGGGGGGTGCAGGGGGGGTTGCAGCCCGACCGACGCGCCCACACCCCCTGCTCACCCCCCCACGCACACACCCCGCACGCAG
CCTTTGTTCCCCTCGCAGCCCCCCCGCACCGCGGGGCACCGCCCCCGGCCGCGCTCCCCTCGCGCACACTGCGGAGCGCACAAAGCCCCGCGCCGCGCCCGCAGCGC
TCACAGCCGCCGGGCAGCGCGGAGCCGCACGCGGCGCTCCCCACGCACACACACACGCACGCACCCCCCGAGCCGCTCCCCCCGCACAAAGGGCCCTCCCGGAGCCC
CTCAAGGCTTTCACGCAGCCACAGAAAAGAAACAAGCCGTCATTAAACCAAGCGCTAATTACAGCCCGGAGGAGAAGGGCCGTCCCGCCCGCTCACCTGTGGGAGTAACG
CGGTCAGTCAGAGCCGGGGCGGGCGGCGCGAGGCGGCGGCGGAGCGGGGCACGGGGCGAAGGCAGCGCGCAGCGACTCCCGCCCGCCGCGCGCTTCGCTTTTTATA
GGGCCGCCGCCGCCGCCGCCTCGCCATAAAAGGAAACTTTCGGAGCGCGCCGCTCTGATTGGCTGCCGCCGCACCTCTCCGCCTCGCCCCGCCCCGCCCCTCGCCCCG
CCCCGCCCCGCCTGGCGCGCGCCCCCCCCCCCCCGCCCCCATCGCTGCACAAAATAATTAAAAAATAAATAAATACAAAATTGGGGGTGGGGAGGGGGGGGAGATGGG
GAGAGTGAAGCAGAACGTGGGGCTCACCTCGACCATGGTAATAGCGATGACTAATACGTAGATGTACTGCCAAGTAGGAAAGTCCCATAAGGTCATGTACTGGGCATAATG
CCAGGCGGGCCATTTACCGTCATTGACGTCAATAGGGGGCGTACTTGGCATATGATACACTTGATGTACTGCCAAGTGGGCAGTTTACCGTAAATACTCCACCCATTGACGT
CAATGGAAAGTCCCTATTGGCGTTACTATGGGAACATACGTCATTATTGACGTCAATGGGCGGGGGTCGTTGGGCGGTCAGCCAGGCGGGCCATTTACCGTAAGTTATGTA
ACGCGGAACTCCATATATGGGCTATGAACTAATGACCCCGTAATTGATTACTATTAATAACTAGTCAATAATCAATGTCGACCCAGGTGGCACTTTTCGGGGAAATGTGCGCG
GAACCCCTATTTGTTTATTTTTCTAAATACATTCAAATATGTATCCGCTCATGAGACAATAACCCTGATAAATGCTTCAATAATATTGAAAAAGGAAGAGTATGAGTATTCAACAT
TTCCGTGTCGCCCTTATTCCCTTTTTTGCGGCATTTTGCCTTCCTGTTTTTGCTCACCCAGAAACGCTGGTGAAAGTAAAAGATGCTGAAGATCAGTTGGGTGCACGAGTGGG
TTACATCGAACTGGATCTCAACAGCGGTAAGATCCTTGAGAGTTTTCGCCCCGAAGAACGTTTTCCAATGATGAGCACTTTTAAAGTTCTGCTATGTGGCGCGGTATTATCCC
GTATTGACGCCGGGCAAGAGCAACTCGGTCGCCGCATACACTATTCTCAGAATGACTTGGTTGAGTACTCACCAGTCACAGAAAAGCATCTTACGGATGGCATGACAGTAAG
AGAATTATGCAGTGCTGCCATAACCATGAGTGATAACACTGCGGCCAACTTACTTCTGACAACGATCGGAGGACCGAAGGAGCTAACCGCTTTTTTGCACAACATGGGGGAT
CATGTAACTCGCCTTGATCGTTGGGAACCGGAGCTGAATGAAGCCATACCAAACGACGAGCGTGACACCACGATGCCTGTAGCAATGGCAACAACGTTGCGCAAACTATTA
ACTGGCGAACTACTTACTCTAGCTTCCCGGCAACAATTAATAGACTGGATGGAGGCGGATAAAGTTGCAGGACCACTTCTGCGCTCGGCCCTTCCGGCTGGCTGGTTTATTG
CTGATAAATCTGGAGCCGGTGAGCGTGGGTCTCGCGGTATCATTGCAGCACTGGGGCCAGATGGTAAGCCCTCCCGTATCGTAGTTATCTACACGACGGGGAGTCAGGCA
ACTATGGATGAACGAAATAGACAGATCGCTGAGATAGGTGCCTCACTGATTAAGCATTGGTAACTGTCAGACCAAGTTTACTCATATATACTTTAGATTGATTTAAAACTTCAT
TTTTAATTTAAAAGGATCTAGGTGAAGATCCTTTTTGATAATCTCATGACCAAAATCCCTTAACGTGAGTTTTCGTTCCACTGAGCGTCAGACCCCGTAGAAAAGATCAAAGGA
TCTTCTTGAGATCCTTTTTTTCTGCGCGTAATCTGCTGCTTGCAAACAAAAAAACCACCGCTACCAGCGGTGGTTTGTTTGCCGGATCAAGAGCTACCAACTCTTTTTCCGAA
GGTAACTGGCTTCAGCAGAGCGCAGATACCAAATACTGTTCTTCTAGTGTAGCCGTAGTTAGGCCACCACTTCAAGAACTCTGTAGCACCGCCTACATACCTCGCTCTGCTA
ATCCTGTTACCAGTGGCTGCTGCCAGTGGCGATAAGTCGTGTCTTACCGGGTTGGACTCAAGACGATAGTTACCGGATAAGGCGCAGCGGTCGGGCTGAACGGGGGGTTC
GTGCACACAGCCCAGCTTGGAGCGAACGACCTACACCGAACTGAGATACCTACAGCGTGAGCTATGAGAAAGCGCCACGCTTCCCGAAGGGAGAAAGGCGGACAGGTATC
CGGTAAGCGGCAGGGTCGGAACAGGAGAGCGCACGAGGGAGCTTCCAGGGGGAAACGCCTGGTATCTTTATAGTCCTGTCGGGTTTCGCCACCTCTGACTTGAGCGTCGA
TTTTTGTGATGCTCGTCAGGGGGGCGGAGCCTATGGAAAAACGCCAGCAACGCGGCCTTTTTACGGTTCCTGGCCTTTTGCTGGCCTTTTGCTCACATGTTCTTTCCTGCGT
TATCCCCTGATTCTGTGGATAACCGTATTACCGCCTTTGAGTGAGCTGATACCGCTCGCCGCAGCCGAACGACCGAGCGCAGCGAGTCAGTGAGCGAGGAAGCGGAAGAG
CGCCCAATACGCAAACCGCCTCTCCCCGCGCGTTGGCCGATTCATTAATGCAGCGGATCCAGACATGATAAGATACATTGATGAGTTTGGACAAACCACAACTAGAATGCAG
TGAAAAAAATGCTTTATTTGTGAAATTTGTGATGCTATTGCTTTATTTGTAACCATTATAAGCTGCAATAAACAAGTTAGCTTTTTGCAAAAGCCTAGGCCTCCAAAAAAGCCTC
CTCACTACTTCTGGAATAGCTCAGAGGCCGAGGCGGCCTCGGCCTCTGCATAAATAAAAAAAATTAGTCAGCCATGGGGCGGAGAATGGGCGGAACTGGGCGGAGTTAGG
GGCGGGATGGGCGGAGTTAGGGGCGGGACTATGGTTGCTGACTAATTGAGATGCGGATCCGCTGGCACGACAGGTTTCCCGACTGGAAAGCGGGCAGTGAGCGCAACGC
AATTAATGTGAGTTAGCTCACTCATTAGGCACCCCAGGCTTTACACTTTATGCTTCCGGCTCGTATGTTGTGTGGAATTGTGAGCGGATAACAATTTCACACAGGAAACAGCT
ATGACCATGATTACGCCAAGCTTGGGCTGCAGGTCGAGGGATCTCCATAAGAGAAGAGGGACAGCTATGACTGGGAGTAGTCAGGAGAGGAGGAAAAATCTGGCTAGTAA
AACATGTAAGGAAAATTTTAGGGATGTTAAAGAAAAAAATAACACAAAACAAAATAT

Supplementary Table 2. Sequence coverage for mCherry-HSP90Beta-AA construct showing 351x coverage.

>mCherry-HSP90B-AA   coverage: 351x

AAAAAAAATCTAACCTCAAGTCAAGGCTTTTCTATGGAATAAGGAATGGACAGCAGGGGGCTGTTTCATATACTGATGACCTCTTTATAGCCAACCTTTGTTCATGGCAGCCA
GCATATGGGCATATGTTGCCAAACTCTAAACCAAATACTCATTCTGATGTTTTAAATGATTTGCCCTCCCATATGTCCTTCCGAGTGAGAGACACAAAAAATTCCAACACACTA
TTGCAATGAAAATAAATTTCCTTTATTAGCCAGAAGTCAGATGCTCAAGGGGCTTCATGATGTCCCCATAATTTTTGGCAGAGGGAAAAAGATTCGAGATCTTCCCTAATCGAC
TTCTTCCATGCGAGACGCATCCTCATCGCCCTCGAGAGGGGGGATCTCATCAGGAACTGCAGCATTGGGTTCCTCTGCTGCCACTTCATCTTCATCAATACCTAGACCTAGC
TTGATCATGCGATAGATGCGGTTGGAGTGGGTCTGGGGATCCTCAAGGGAAAAGCCAGAAGATAGCAGGGCGGTTTCAAACAGCAGCACCACCAGGTCCTTAACTGCCTTA
TCATTCTTGTCGGCCTCAGCCTTCTGCCGCAGCGTCTCCACAATGGGGTGGTCAGGGTTGATCTCCAGGTGCTTTTTGGCCATCATATAGCCCATGGTGGAGTTGTCCCGAA
GTGCCTGGGCTTTCATGATCCGCTCCATATTGGCTGTCCAGCCGTAGGTGCTGGTCACAATGCAGCAAGGTGAAGACACAAGTCTATTGGAGATTGTCACCTTCTCAACCTT
CTTATCTAAGATTTCTTTCATGAGCTTGCAGAGGTTCTCAAACTTTGCCTTGCTCTCTTCCATCTTCTTCTTCTCCTCCTCATCCTCAGGCAGCTCCAGACCCTCCTTGGTAAC
TGAGACCAGGCTCTTCCCATCAAATTCCTTGAGCTGCTGCACACAGTACTCGTCAATGGGCTCGGTCATATATACCACCTCGAAGCCCCGTTTCCGCACTCGCTCCACAAAA
GCTGAGTTGGCCACCTGCTCTTTGCTCTCACCAGTGATGTAATAGATGGACTTCTGTGTCTCCTTCATGCGAGAAACATACTCTGACAGAGATGTCATCTCATCTCCAGACTG
GGAGGTATGATAGCGCAGCAGCTCAGACAGGCGGCGGCGGTTAGTGGAGTCTTCGTGGATTCCAAGCTTGAGATTTTTAGAGAATGCCTCATAGAATTTCTTGTAATTCTCC
TTGTCTTCTGCCAGCTCAGAGAAGAGCTCAAGGCACTTCTTAACAATGTTTTTGCGAATGACTTTCAAGATTTTGCTCTGCTGGAGCATTTCTCGGGAGATGTTCAGGGGCAG
ATCCTCAGAGTCAACCACACCACGGATAAAATTGAGATACTCTGGTATCAACTCATCACAGCTGTCCATGATGAACACACGGCGGACATAGAGTTTGATGTTGTTCTTTTTCT
TCTTGTTCTCAAAAAGGTCAAAGGGAGCCCGACGAGGAATAAATAGCAATGCCCTGAATTCCAACTGACCTTCTACAGAAAAGTGCTTGACTGCCAAGTGGTCTTCCCAGTC
ATTAGTGAGGCTCTTGTAGAATTCTCCATACTCCTCTTGGGTGATGTCATCAGGGTTTCTGGTCCAAATAGGCTTGGTCTTGTTTAGTTCTTCCTGATCAATGTATTTCTCTTTG
ATCTTCTTAGTTTTCTTCTTCTTATCCTTACCGCTGTCATCCTCCTCATCGGCACCCACATCTTCGATCTTGGGCTTTTCTTCATCATCTTTATCTTCCTCTTCTTTCTCACCTTT
CTCTTCCTCTGCCTCATCATCGGCAATTTCCTTCTCTCGTTCCTTCTCCAAATAAAGGGTGATGGGATAGCCTATGAACTGAGAATGCTTCTTCACTACTTCTTTGACCCGCCT
CTCTTCTAGGTACTCTGTCTGATCTTCTTTAAGATGGAGGATCACTTTGGTACCCCTGCCAATGGGCTCACCATGGTCAGCACGCACAGTGAAGGAACCTCCAGCAGAAGAC
TCCCAAGCATACTGTTCATCATCGTTGTGCTTTGTGATCACAACCACTTTCTCTGCCACCAAGTAGGCAGAATAAAAGCCAACACCAAACTGCCCAATCATGGAGATGTCTGC
ACCAGCCTGAAGAGCCTCCATGAATGCTTTAGTACCAGACTTGGCAATGGTTCCCAAATTATTTATGAGATCAGCTTTGGTCATGCCAATGCCTGTGTCAACCAAAGTCAGGG
TACGTTCCTGAGGGTTGGGGATGATGTCAATTTTCAGCTCTTTACCACTGTCCAACTTCGAAGGGTCTGTCAGGCTCTCATAGCGAATCTTGTCCAAGGCATCAGAAGCATTA
GAGATCAACTCCCGAAGGAAAATCTCCTTGTTGGAATAGAAGGTATTGATGATGAGGGACATGAGTTGGGCAATTTCTGCCTGAAAGGCAAAAGTCTCCACCTCCTCCTCTC
CATGGTGCACTTCCTCTGGCATCGGTCCGGACTTGTACAGCTCGTCCATGCCGCCGGTGGAGTGGCGGCCCTCGGCGCGTTCGTACTGTTCCACGATGGTGTAGTCCTCG
TTGTGGGAGGTGATGTCCAACTTGATGTTGACGTTGTAGGCGCCGGGCAGCTGCACGGGCTTCTTGGCCTTGTAGGTGGTCTTGACCTCAGCGTCGTAGTGGCCGCCGTC
CTTCAGCTTCAGCCTCTGCTTGATCTCGCCCTTCAGGGCGCCGTCCTCGGGGTACATCCGCTCGGAGGAGGCCTCCCAGCCCATGGTCTTCTTCTGCATTACGGGGCCGT
CGGAGGGGAAGTTGGTGCCGCGCAGCTTCACCTTGTAGATGAACTCGCCGTCCTGCAGGGAGGAGTCCTGGGTCACGGTCACCACGCCGCCGTCCTCGAAGTTCATCAC
GCGCTCCCACTTGAAGCCCTCGGGGAAGGACAGCTTCAAGTAGTCGGGGATGTCGGCGGGGTGCTTCACGTAGGCCTTGGAGCCGTACATGAACTGAGGGGACAGGATG
TCCCAGGCGAAGGGCAGGGGGCCACCCTTGGTCACCTTCAGCTTGGCGGTCTGGGTGCCCTCGTAGGGGCGGCCCTCGCCCTCGCCCTCGATCTCGAACTCGTGGCCGT
TCACGGAGCCCTCCATGTGCACCTTGAAGCGCATGAACTCCTTGATGATGGCCATGTTATCCTCCTCGCCCTTGCTCACCATGGTGGCGCTAGAATTCTTTGCCAAAATGAT
GAGACAGCACAATAACCAGCACGTTGCCCAGGAGCTGTAGGAAAAAGAAGAAGGCATGAACATGGTTAGCAGAGGCTCTAGAGCCGCCGGTCACACGCCAGAAGCCGAAC
CCCGCCCTGCCCCGTCCCCCCCGAAGGCAGCCGTCCCCCCGCGGACAGCCCCGAGGCTGGAGAGGGAGAAGGGGACGGCGGCGCGGCGACGCACGAAGGCCCTCCCC
GCCCATTTCCTTCCTGCCGGCGCCGCACCGCTTCGCCCCGCGCCCGCTAGAGGGGGTGCGGCGGCGCCTCCCAGATTTCGGCTCCGCACAGATTTGGGACAAAGGAAGT
CCCTGCGCCCTCTCGCACGATTACCATAAAAGGCAATGGCTGCGGCTCGCCGCGCCTCGACAGCCGCCGGCGCTCCGGGGGCCGCCGCGCCCCTCCCCCGAGCCCTCC
CCGGCCCGAGGCGGCCCCGCCCCGCCCGGCACCCCCACCTGCCGCCACCCCCCGCCCGGCACGGCGAGCCCCGCGCCACGCCCCGTACGGAGCCCCGCACCCGAAG
CCGGGCCGTGCTCAGCAACTCGGGGAGGGGGGTGCAGGGGGGGTTGCAGCCCGACCGACGCGCCCACACCCCCTGCTCACCCCCCCACGCACACACCCCGCACGCAG
CCTTTGTTCCCCTCGCAGCCCCCCCCGCACCGCGGGGCACCGCCCCCGGCCGCGCTCCCCTCGCGCACACTGCGGAGCGCACAAAGCCCCGCGCCGCGCCCGCAGCGC
TCACAGCCGCCGGGCAGCGCGGAGCCGCACGCGGCGCTCCCCACGCACACACACACGCACGCACCCCCCGAGCCGCTCCCCCCGCACAAAGGGCCCTCCCGGAGCCC
CTCAAGGCTTTCACGCAGCCACAGAAAAGAAACAAGCCGTCATTAAACCAAGCGCTAATTACAGCCCGGAGGAGAAGGGCCGTCCCGCCCGCTCACCTGTGGGAGTAACG
CGGTCAGTCAGAGCCGGGGCGGGCGGCGCGAGGCGGCGGCGGAGCGGGGCACGGGGCGAAGGCAGCGCGCAGCGACTCCCGCCCGCCGCGCGCTTCGCTTTTTATA
GGGCCGCCGCCGCCGCCGCCTCGCCATAAAAGGAAACTTTCGGAGCGCGCCGCTCTGATTGGCTGCCGCCGCACCTCTCCGCCTCGCCCCGCCCCGCCCCTCGCCCCG
CCCCGCCCCGCCTGGCGCGCGCCCCCCCCCCCCGCCCCCATCGCTGCACAAAATAATTAAAAAATAAATAAATACAAAATTGGGGGTGGGGAGGGGGGGGAGATGGGG
AGAGTGAAGCAGAACGTGGGGCTCACCTCGACCATGGTAATAGCGATGACTAATACGTAGATGTACTGCCAAGTAGGAAAGTCCCATAAGGTCATGTACTGGGCATAATGC
CAGGCGGGCCATTTACCGTCATTGACGTCAATAGGGGGCGTACTTGGCATATGATACACTTGATGTACTGCCAAGTGGGCAGTTTACCGTAAATACTCCACCCATTGACGTC
AATGGAAAGTCCCTATTGGCGTTACTATGGGAACATACGTCATTATTGACGTCAATGGGCGGGGGTCGTTGGGCGGTCAGCCAGGCGGGCCATTTACCGTAAGTTATGTAA
CGCGGAACTCCATATATGGGCTATGAACTAATGACCCCGTAATTGATTACTATTAATAACTAGTCAATAATCAATGTCGACCCAGGTGGCACTTTTCGGGGAAATGTGCGCGG
AACCCCTATTTGTTTATTTTTCTAAATACATTCAAATATGTATCCGCTCATGAGACAATAACCCTGATAAATGCTTCAATAATATTGAAAAAGGAAGAGTATGAGTATTCAACATT
TCCGTGTCGCCCTTATTCCCTTTTTTGCGGCATTTTGCCTTCCTGTTTTTGCTCACCCAGAAACGCTGGTGAAAGTAAAAGATGCTGAAGATCAGTTGGGTGCACGAGTGGGT
TACATCGAACTGGATCTCAACAGCGGTAAGATCCTTGAGAGTTTTCGCCCCGAAGAACGTTTTCCAATGATGAGCACTTTTAAAGTTCTGCTATGTGGCGCGGTATTATCCCG
TATTGACGCCGGGCAAGAGCAACTCGGTCGCCGCATACACTATTCTCAGAATGACTTGGTTGAGTACTCACCAGTCACAGAAAAGCATCTTACGGATGGCATGACAGTAAGA
GAATTATGCAGTGCTGCCATAACCATGAGTGATAACACTGCGGCCAACTTACTTCTGACAACGATCGGAGGACCGAAGGAGCTAACCGCTTTTTTGCACAACATGGGGGATC
ATGTAACTCGCCTTGATCGTTGGGAACCGGAGCTGAATGAAGCCATACCAAACGACGAGCGTGACACCACGATGCCTGTAGCAATGGCAACAACGTTGCGCAAACTATTAA
CTGGCGAACTACTTACTCTAGCTTCCCGGCAACAATTAATAGACTGGATGGAGGCGGATAAAGTTGCAGGACCACTTCTGCGCTCGGCCCTTCCGGCTGGCTGGTTTATTG
CTGATAAATCTGGAGCCGGTGAGCGTGGGTCTCGCGGTATCATTGCAGCACTGGGGCCAGATGGTAAGCCCTCCCGTATCGTAGTTATCTACACGACGGGGAGTCAGGCA
ACTATGGATGAACGAAATAGACAGATCGCTGAGATAGGTGCCTCACTGATTAAGCATTGGTAACTGTCAGACCAAGTTTACTCATATATACTTTAGATTGATTTAAAACTTCAT
TTTTAATTTAAAAGGATCTAGGTGAAGATCCTTTTTGATAATCTCATGACCAAAATCCCTTAACGTGAGTTTTCGTTCCACTGAGCGTCAGACCCCGTAGAAAAGATCAAAGGA
TCTTCTTGAGATCCTTTTTTTCTGCGCGTAATCTGCTGCTTGCAAACAAAAAAACCACCGCTACCAGCGGTGGTTTGTTTGCCGGATCAAGAGCTACCAACTCTTTTTCCGAA
GGTAACTGGCTTCAGCAGAGCGCAGATACCAAATACTGTTCTTCTAGTGTAGCCGTAGTTAGGCCACCACTTCAAGAACTCTGTAGCACCGCCTACATACCTCGCTCTGCTA
ATCCTGTTACCAGTGGCTGCTGCCAGTGGCGATAAGTCGTGTCTTACCGGGTTGGACTCAAGACGATAGTTACCGGATAAGGCGCAGCGGTCGGGCTGAACGGGGGGTTC
GTGCACACAGCCCAGCTTGGAGCGAACGACCTACACCGAACTGAGATACCTACAGCGTGAGCTATGAGAAAGCGCCACGCTTCCCGAAGGGAGAAAGGCGGACAGGTATC
CGGTAAGCGGCAGGGTCGGAACAGGAGAGCGCACGAGGGAGCTTCCAGGGGGAAACGCCTGGTATCTTTATAGTCCTGTCGGGTTTCGCCACCTCTGACTTGAGCGTCGA
TTTTTGTGATGCTCGTCAGGGGGGCGGAGCCTATGGAAAAACGCCAGCAACGCGGCCTTTTTACGGTTCCTGGCCTTTTGCTGGCCTTTTGCTCACATGTTCTTTCCTGCGT
TATCCCCTGATTCTGTGGATAACCGTATTACCGCCTTTGAGTGAGCTGATACCGCTCGCCGCAGCCGAACGACCGAGCGCAGCGAGTCAGTGAGCGAGGAAGCGGAAGAG
CGCCCAATACGCAAACCGCCTCTCCCCGCGCGTTGGCCGATTCATTAATGCAGCGGATCCAGACATGATAAGATACATTGATGAGTTTGGACAAACCACAACTAGAATGCAG
TGAAAAAAATGCTTTATTTGTGAAATTTGTGATGCTATTGCTTTATTTGTAACCATTATAAGCTGCAATAAACAAGTTAGCTTTTTGCAAAAGCCTAGGCCTCCAAAAAAGCCTC
CTCACTACTTCTGGAATAGCTCAGAGGCCGAGGCGGCCTCGGCCTCTGCATAAATAAAAAAAATTAGTCAGCCATGGGGCGGAGAATGGGCGGAACTGGGCGGAGTTAGG
GGCGGGATGGGCGGAGTTAGGGGCGGGACTATGGTTGCTGACTAATTGAGATGCGGATCCGCTGGCACGACAGGTTTCCCGACTGGAAAGCGGGCAGTGAGCGCAACGC
AATTAATGTGAGTTAGCTCACTCATTAGGCACCCCAGGCTTTACACTTTATGCTTCCGGCTCGTATGTTGTGTGGAATTGTGAGCGGATAACAATTTCACACAGGAAACAGCT
ATGACCATGATTACGCCAAGCTTGGGCTGCAGGTCGAGGGATCTCCATAAGAGAAGAGGGACAGCTATGACTGGGAGTAGTCAGGAGAGGAGGAAAAATCTGGCTAGTAA
AACATGTAAGGAAAATTTTAGGGATGTTAAAGAAAAAAATAACACAAAACAAAATAT

Supplementary Table 3. Sequence coverage for mCherry-HSP90Beta-EE construct showing 480x coverage.

>mCherry-HSP90B-EE coverage: 480x

AAAAAAAATCTAACCTCAAGTCAAGGCTTTTCTATGGAATAAGGAATGGACAGCAGGGGGCTGTTTCATATACTGATGACCTCTTTATAGCCAACCTTTGTTCATGGCAGCCA
GCATATGGGCATATGTTGCCAAACTCTAAACCAAATACTCATTCTGATGTTTTAAATGATTTGCCCTCCCATATGTCCTTCCGAGTGAGAGACACAAAAAATTCCAACACACTA
TTGCAATGAAAATAAATTTCCTTTATTAGCCAGAAGTCAGATGCTCAAGGGGCTTCATGATGTCCCCATAATTTTTGGCAGAGGGAAAAAGATTGAGATCTTCCCTAATCGACT
TCTTCCATGCGAGACGCATCCTCATCGCCCTCGAGAGGGGGGATCTCATCAGGAACTGCAGCATTGGGTTCCTCTGCTGCCACTTCATCTTCATCAATACCTAGACCTAGCT
TGATCATGCGATAGATGCGGTTGGAGTGGGTCTGGGGATCCTCAAGGGAAAAGCCAGAAGATAGCAGGGCGGTTTCAAACAGCAGCACCACCAGGTCCTTAACTGCCTTAT
CATTCTTGTCGGCCTCAGCCTTCTGCCGCAGCGTCTCCACAATGGGGTGGTCAGGGTTGATCTCCAGGTGCTTTTTGGCCATCATATAGCCCATGGTGGAGTTGTCCCGAA
GTGCCTGGGCTTTCATGATCCGCTCCATATTGGCTGTCCAGCCGTAGGTGCTGGTCACAATGCAGCAAGGTGAAGACACAAGTCTATTGGAGATTGTCACCTTCTCAACCTT
CTTATCTAAGATTTCTTTCATGAGCTTGCAGAGGTTCTCAAACTTTGCCTTGCTCTCTTCCATCTTCTTCTTCTCCTCCTCATCCTCAGGCAGCTCCAGACCCTCCTTGGTAAC
TGAGACCAGGCTCTTCCCATCAAATTCCTTGAGCTGCTGCACACAGTACTCGTCAATGGGCTCGGTCATATATACCACCTCGAAGCCCCGTTTCCGCACTCGCTCCACAAAA
GCTGAGTTGGCCACCTGCTCTTTGCTCTCACCAGTGATGTAATAGATGGACTTCTGTGTCTCCTTCATGCGAGAAACATACTCTGACAGAGATGTCATCTCATCTCCAGACTG
GGAGGTATGATAGCGCAGCAGCTCAGACAGGCGGCGGCGGTTAGTGGAGTCTTCGTGGATTCCAAGCTTGAGATTTTTAGAGAATGCCTCATAGAATTTCTTGTAATTCTCC
TTGTCTTCTGCCAGCTCAGAGAAGAGCTCAAGGCACTTCTTAACAATGTTTTTGCGAATGACTTTCAAGATTTTGCTCTGCTGGAGCATTTCTCGGGAGATGTTCAGGGGCAG
ATCCTCAGAGTCAACCACACCACGGATAAAATTGAGATACTCTGGTATCAACTCATCACAGCTGTCCATGATGAACACACGGCGGACATAGAGTTTGATGTTGTTCTTTTTCT
TCTTGTTCTCAAAAAGGTCAAAGGGAGCCCGACGAGGAATAAATAGCAATGCCCTGAATTCCAACTGACCTTCTACAGAAAAGTGCTTGACTGCCAAGTGGTCTTCCCAGTC
ATTAGTGAGGCTCTTGTAGAATTCTCCATACTCCTCTTGGGTGATGTCATCAGGGTTTCTGGTCCAAATAGGCTTGGTCTTGTTTAGTTCTTCCTGATCAATGTATTTCTCTTTG
ATCTTCTTAGTTTTCTTCTTCTTATCCTTACCGCTGTCATCCTCCTCATCCTCACCCACATCTTCGATCTTGGGCTTTTCTTCATCATCTTTATCTTCCTCTTCTTTCTCACCTTTC
TCTTCCTCTGCCTCATCATCCTCAATTTCCTTCTCTCGTTCCTTCTCCAAATAAAGGGTGATGGGATAGCCTATGAACTGAGAATGCTTCTTCACTACTTCTTTGACCCGCCTC
TCTTCTAGGTACTCTGTCTGATCTTCTTTAAGATGGAGGATCACTTTGGTACCCCTGCCAATGGGCTCACCATGGTCAGCACGCACAGTGAAGGAACCTCCAGCAGAAGACT
CCCAAGCATACTGTTCATCATCGTTGTGCTTTGTGATCACAACCACTTTCTCTGCCACCAAGTAGGCAGAATAAAAGCCAACACCAAACTGCCCAATCATGGAGATGTCTGCA
CCAGCCTGAAGAGCCTCCATGAATGCTTTAGTACCAGACTTGGCAATGGTTCCCAAATTATTTATGAGATCAGCTTTGGTCATGCCAATGCCTGTGTCAACCAAAGTCAGGGT
ACGTTCCTGAGGGTTGGGGATGATGTCAATTTTCAGCTCTTTACCACTGTCCAACTTCGAAGGGTCTGTCAGGCTCTCATAGCGAATCTTGTCCAAGGCATCAGAAGCATTA
GAGATCAACTCCCGAAGGAAAATCTCCTTGTTGGAATAGAAGGTATTGATGATGAGGGACATGAGTTGGGCAATTTCTGCCTGAAAGGCAAAAGTCTCCACCTCCTCCTCTC
CATGGTGCACTTCCTCTGGCATCGGTCCGGACTTGTACAGCTCGTCCATGCCGCCGGTGGAGTGGCGGCCCTCGGCGCGTTCGTACTGTTCCACGATGGTGTAGTCCTCG
TTGTGGGAGGTGATGTCCAACTTGATGTTGACGTTGTAGGCGCCGGGCAGCTGCACGGGCTTCTTGGCCTTGTAGGTGGTCTTGACCTCAGCGTCGTAGTGGCCGCCGTC
CTTCAGCTTCAGCCTCTGCTTGATCTCGCCCTTCAGGGCGCCGTCCTCGGGGTACATCCGCTCGGAGGAGGCCTCCCAGCCCATGGTCTTCTTCTGCATTACGGGGCCGT
CGGAGGGGAAGTTGGTGCCGCGCAGCTTCACCTTGTAGATGAACTCGCCGTCCTGCAGGGAGGAGTCCTGGGTCACGGTCACCACGCCGCCGTCCTCGAAGTTCATCAC
GCGCTCCCACTTGAAGCCCTCGGGGAAGGACAGCTTCAAGTAGTCGGGGATGTCGGCGGGGTGCTTCACGTAGGCCTTGGAGCCGTACATGAACTGAGGGGACAGGATG
TCCCAGGCGAAGGGCAGGGGGCCACCCTTGGTCACCTTCAGCTTGGCGGTCTGGGTGCCCTCGTAGGGGCGGCCCTCGCCCTCGCCCTCGATCTCGAACTCGTGGCCGT
TCACGGAGCCCTCCATGTGCACCTTGAAGCGCATGAACTCCTTGATGATGGCCATGTTATCCTCCTCGCCCTTGCTCACCATGGTGGCGCTAGAATTCTTTGCCAAAATGAT
GAGACAGCACAATAACCAGCACGTTGCCCAGGAGCTGTAGGAAAAAGAAGAAGGCATGAACATGGTTAGCAGAGGCTCTAGAGCCGCCGGTCACACGCCAGAAGCCGAAC
CCCGCCCTGCCCCGTCCCCCCCGAAGGCAGCCGTCCCCCCGCGGACAGCCCCGAGGCTGGAGAGGGAGAAGGGGACGGCGGCGCGGCGACGCACGAAGGCCCTCCCC
GCCCATTTCCTTCCTGCCGGCGCCGCACCGCTTCGCCCCGCGCCCGCTAGAGGGGGTGCGGCGGCGCCTCCCAGATTTCGGCTCCGCACAGATTTGGGACAAAGGAAGT
CCCTGCGCCCTCTCGCACGATTACCATAAAAGGCAATGGCTGCGGCTCGCCGCGCCTCGACAGCCGCCGGCGCTCCGGGGGCCGCCGCGCCCCTCCCCCGAGCCCTCC
CCGGCCCGAGGCGGCCCCGCCCCGCCCGGCACCCCCACCTGCCGCCACCCCCCGCCCGGCACGGCGAGCCCCGCGCCACGCCCCGTACGGAGCCCCGCACCCGAAG
CCGGGCCGTGCTCAGCAACTCGGGGAGGGGGGTGCAGGGGGGGTTGCAGCCCGACCGACGCGCCCACACCCCCTGCTCACCCCCCCACGCACACACCCCGCACGCAG
CCTTTGTTCCCCTCGCAGCCCCCCCCGCACCGCGGGGCACCGCCCCCGGCCGCGCTCCCCTCGCGCACACTGCGGAGCGCACAAAGCCCCGCGCCGCGCCCGCAGCGC
TCACAGCCGCCGGGCAGCGCGGAGCCGCACGCGGCGCTCCCCACGCACACACACACGCACGCACCCCCCGAGCCGCTCCCCCCGCACAAAGGGCCCTCCCGGAGCCC
CTCAAGGCTTTCACGCAGCCACAGAAAAGAAACAAGCCGTCATTAAACCAAGCGCTAATTACAGCCCGGAGGAGAAGGGCCGTCCCGCCCGCTCACCTGTGGGAGTAACG
CGGTCAGTCAGAGCCGGGGCGGGCGGCGCGAGGCGGCGGCGGAGCGGGGCACGGGGCGAAGGCAGCGCGCAGCGACTCCCGCCCGCCGCGCGCTTCGCTTTTTATA
GGGCCGCCGCCGCCGCCGCCTCGCCATAAAAGGAAACTTTCGGAGCGCGCCGCTCTGATTGGCTGCCGCCGCACCTCTCCGCCTCGCCCCGCCCCGCCCCTCGCCCCG
CCCCGCCCCGCCTGGCGCGCGCCCCCCCCCCCCCCGCCCCCATCGCTGCACAAAATAATTAAAAAATAAATAAATACAAAATTGGGGGTGGGGAGGGGGGGGAGATGG
GGAGAGTGAAGCAGAACGTGGGGCTCACCTCGACCATGGTAATAGCGATGACTAATACGTAGATGTACTGCCAAGTAGGAAAGTCCCATAAGGTCATGTACTGGGCATAAT
GCCAGGCGGGCCATTTACCGTCATTGACGTCAATAGGGGGCGTACTTGGCATATGATACACTTGATGTACTGCCAAGTGGGCAGTTTACCGTAAATACTCCACCCATTGACG
TCAATGGAAAGTCCCTATTGGCGTTACTATGGGAACATACGTCATTATTGACGTCAATGGGCGGGGGTCGTTGGGCGGTCAGCCAGGCGGGCCATTTACCGTAAGTTATGT
AACGCGGAACTCCATATATGGGCTATGAACTAATGACCCCGTAATTGATTACTATTAATAACTAGTCAATAATCAATGTCGACCCAGGTGGCACTTTTCGGGGAAATGTGCGC
GGAACCCCTATTTGTTTATTTTTCTAAATACATTCAAATATGTATCCGCTCATGAGACAATAACCCTGATAAATGCTTCAATAATATTGAAAAAGGAAGAGTATGAGTATTCAAC
ATTTCCGTGTCGCCCTTATTCCCTTTTTTGCGGCATTTTGCCTTCCTGTTTTTGCTCACCCAGAAACGCTGGTGAAAGTAAAAGATGCTGAAGATCAGTTGGGTGCACGAGTG
GGTTACATCGAACTGGATCTCAACAGCGGTAAGATCCTTGAGAGTTTTCGCCCCGAAGAACGTTTTCCAATGATGAGCACTTTTAAAGTTCTGCTATGTGGCGCGGTATTATC
CCGTATTGACGCCGGGCAAGAGCAACTCGGTCGCCGCATACACTATTCTCAGAATGACTTGGTTGAGTACTCACCAGTCACAGAAAAGCATCTTACGGATGGCATGACAGTA
AGAGAATTATGCAGTGCTGCCATAACCATGAGTGATAACACTGCGGCCAACTTACTTCTGACAACGATCGGAGGACCGAAGGAGCTAACCGCTTTTTTGCACAACATGGGG
GATCATGTAACTCGCCTTGATCGTTGGGAACCGGAGCTGAATGAAGCCATACCAAACGACGAGCGTGACACCACGATGCCTGTAGCAATGGCAACAACGTTGCGCAAACTA
TTAACTGGCGAACTACTTACTCTAGCTTCCCGGCAACAATTAATAGACTGGATGGAGGCGGATAAAGTTGCAGGACCACTTCTGCGCTCGGCCCTTCCGGCTGGCTGGTTTA
TTGCTGATAAATCTGGAGCCGGTGAGCGTGGGTCTCGCGGTATCATTGCAGCACTGGGGCCAGATGGTAAGCCCTCCCGTATCGTAGTTATCTACACGACGGGGAGTCAG
GCAACTATGGATGAACGAAATAGACAGATCGCTGAGATAGGTGCCTCACTGATTAAGCATTGGTAACTGTCAGACCAAGTTTACTCATATATACTTTAGATTGATTTAAAACTT
CATTTTTAATTTAAAAGGATCTAGGTGAAGATCCTTTTTGATAATCTCATGACCAAAATCCCTTAACGTGAGTTTTCGTTCCACTGAGCGTCAGACCCCGTAGAAAAGATCAAA
GGATCTTCTTGAGATCCTTTTTTTCTGCGCGTAATCTGCTGCTTGCAAACAAAAAAACCACCGCTACCAGCGGTGGTTTGTTTGCCGGATCAAGAGCTACCAACTCTTTTTCC
GAAGGTAACTGGCTTCAGCAGAGCGCAGATACCAAATACTGTTCTTCTAGTGTAGCCGTAGTTAGGCCACCACTTCAAGAACTCTGTAGCACCGCCTACATACCTCGCTCTG
CTAATCCTGTTACCAGTGGCTGCTGCCAGTGGCGATAAGTCGTGTCTTACCGGGTTGGACTCAAGACGATAGTTACCGGATAAGGCGCAGCGGTCGGGCTGAACGGGGGG
TTCGTGCACACAGCCCAGCTTGGAGCGAACGACCTACACCGAACTGAGATACCTACAGCGTGAGCTATGAGAAAGCGCCACGCTTCCCGAAGGGAGAAAGGCGGACAGGT
ATCCGGTAAGCGGCAGGGTCGGAACAGGAGAGCGCACGAGGGAGCTTCCAGGGGGAAACGCCTGGTATCTTTATAGTCCTGTCGGGTTTCGCCACCTCTGACTTGAGCGT
CGATTTTTGTGATGCTCGTCAGGGGGGCGGAGCCTATGGAAAAACGCCAGCAACGCGGCCTTTTTACGGTTCCTGGCCTTTTGCTGGCCTTTTGCTCACATGTTCTTTCCTG
CGTTATCCCCTGATTCTGTGGATAACCGTATTACCGCCTTTGAGTGAGCTGATACCGCTCGCCGCAGCCGAACGACCGAGCGCAGCGAGTCAGTGAGCGAGGAAGCGGAA
GAGCGCCCAATACGCAAACCGCCTCTCCCCGCGCGTTGGCCGATTCATTAATGCAGCGGATCCAGACATGATAAGATACATTGATGAGTTTGGACAAACCACAACTAGAATG
CAGTGAAAAAAATGCTTTATTTGTGAAATTTGTGATGCTATTGCTTTATTTGTAACCATTATAAGCTGCAATAAACAAGTTAGCTTTTTGCAAAAGCCTAGGCCTCCAAAAAAGC
CTCCTCACTACTTCTGGAATAGCTCAGAGGCCGAGGCGGCCTCGGCCTCTGCATAAATAAAAAAAATTAGTCAGCCATGGGGCGGAGAATGGGCGGAACTGGGCGGAGTT
AGGGGCGGGATGGGCGGAGTTAGGGGCGGGACTATGGTTGCTGACTAATTGAGATGCGGATCCGCTGGCACGACAGGTTTCCCGACTGGAAAGCGGGCAGTGAGCGCAA
CGCAATTAATGTGAGTTAGCTCACTCATTAGGCACCCCAGGCTTTACACTTTATGCTTCCGGCTCGTATGTTGTGTGGAATTGTGAGCGGATAACAATTTCACACAGGAAACA
GCTATGACCATGATTACGCCAAGCTTGGGCTGCAGGTCGAGGGATCTCCATAAGAGAAGAGGGACAGCTATGACTGGGAGTAGTCAGGAGAGGAGGAAAAATCTGGCTAG
TAAAACATGTAAGGAAAATTTTAGGGATGTTAAAGAAAAAAATAACACAAAACAAAATAT

Supplementary Table 4. Summary of Molecular Dynamics Simulations for HSP90β Pentameric Assemblies

Simulation no.	Form of HSP90β in the pentameric assembly	Ligand	MD simulation (each replica) run time (ns)	No. of replicas	Total simulation time
1.	Ser226Ser255 (WT)	ATP	100	3	300 ns
2.	Glu226Glu255 (EE)	ATP	100	3	300 ns
3.	Ala226Ala255 (AA)	ATP	100	3	300 ns
4.	Ser226Ser255 (WT)	ADP	100	3	300 ns
5.	Glu226Glu255 (EE)	ADP	100	3	300 ns
6.	Ala226Ala255 (AA)	ADP	100	3	300 ns

Supplementary Table 5. Reliability and reproducibility checklist for molecular dynamics

**Reliability and reproducibility checklist for molecular dynamics simulations** ***All boxes must be marked YES by acceptance unless an N/A option is available**	**Yes**	**N/A**	**Response (Please state where this information can be found in the text)**
**1. Convergence of simulations and analysis**			
1a. Is an evaluation presented in the text to show that the property being measured has equilibrated in the simulations (*e.g.* time-course analysis)?	☒		Refer to section “Computational analyses” under Methods.
1b. Then, is it described in the text how simulations are split into equilibration and production runs and how much data were analyzed from production runs?	☒		Refer to section “Computational analyses”, Methods.
1c. Are there at least 3 simulations per simulation condition with statistical analysis?	☒		Refer to Methods, Results and Figure Legends
1d. Is evidence provided in the text that the simulation results presented are independent of initial configuration?	☒		Yes, the information is provided under the text under “Results”.
**2. Connection to experiments**			
2a. Are calculations provided that can connect to experiments (*e.g.* loss or gain in function from mutagenesis, binding assays, NMR chemical shifts, J-couplings, SAXS curves, interaction distances or FRET distances, structure factors, diffusion coefficients, bulk modulus and other mechanical properties, *etc.*)?	☐		Not applicable
**3. Method choice**			
3a. Is it described in the text what force field and water model are used and why?	☒		See “Computational analyses”, Methods
3b. Do simulations contain membranes, membrane proteins, intrinsically disordered proteins, glycans, nucleic acids, polymers, or cryptic ligand binding?	☐	☒	Response not needed if **N/A**
If 3b is **YES**, are enhanced sampling methods used?	☐	☐	Response not needed if **N/A**
If enhanced sampling methods are used, are the convergence criteria clearly stated?	☐		
If 3b is **YES**, is it explained in the text why or why not enhanced sampling methods are used?	☐		
**4. Code and reproducibility**			
4a. Is a table provided describing the system setup, such as simulation box dimensions, total number of atoms, total number of water molecules, salt concentration, lipid composition (number of molecules and type)?	☒		The information is provided as SID report in pdf format deposited in Zenodo [https://doi.org/ 10.5281/zenodo.10800912]
4b. Is it described in the text what simulation and analysis software and which versions are used?	☒		The information is provided in the Methods section “Computational analysis”
4c. Are initial coordinate and simulation input files and a coordinate file of the final output provided as supplementary files or in a public repository?	☒		All the input and output files are deposited in Zenodo [https://doi.org/ 10.5281/zenodo.10800912].
4d. Is there custom code or custom force field parameters?	☐	☒	Response not needed if **N/A**
If **YES**, are they provided as supplementary profiles or in a public repository?	☐		

Supplementary Note 1: Chemical synthesis and compound characterization

General methods: All commercial chemicals and solvents were reagent grade and used without further purification. The identity and purity of each product was characterized by MS, HPLC, TLC, and NMR. $^{1}H/^{13}C$ NMR spectra were recorded on either a Bruker 500 or 600 MHz instrument. Chemical shifts are reported in δ values in ppm downfield from TMS as the internal standard. ^{1}H data are reported as follows: chemical shift, multiplicity (s = singlet, d = doublet, t = triplet, q = quartet, br = broad, m = multiplet), coupling constant (Hz), integration. ^{13}C chemical shifts are reported in δ values in ppm downfield from TMS as the internal standard. High resolution mass spectra were recorded on a Waters LCT Premier system. Low resolution mass spectra were obtained on Waters Acquity Ultra Performance LC with electrospray ionization and SQ detector. Purity of target compounds has been determined to be >95% by LC/MS on a Waters Autopurification system with PDA, MicroMass ZQ and ELSD detector and a reversed phase column (Waters X-Bridge C18, 4.6 x 150 mm, 5 μm) eluted with water/acetonitrile gradients, containing 0.1% TFA. Column chromatography was performed using 230-400 mesh silica gel. Analytical thin layer chromatography was performed on 250 μM silica gel F_{254} plates. Preparative thin layer chromatography was performed on 1000 μM silica gel F_{254} plates. Flash chromatography was performed using CombiFlash®Rf instrument.

Synthesis of PU-800CW

2-(3-(6-Amino-8-(6-iodobenzo[d][1,3]dioxol-5-ylthio)-9H-purin-9-yl)hexyl)isoindoline-1,3-dione [2]. To a solution of **1** (50 mg, 0.121 mmol) in DMF (2 mL) was added Cs_2CO_3 (59 mg, 0.182 mmol) and N-(6-bromohexyl)-phthalimide (188 mg, 0.605 mmol) and the mixture was stirred at rt for 6 hr. Solvent was removed under reduced pressure and the resulting residue was purified by preparatory TLC (CH_2Cl_2:MeOH:AcOH, 300:2:4) to give 50 mg (64%) of **6**. ^{1}H NMR (600 MHz, $CDCl_3$) δ 8.27 (s, 1H), 7.82-7.83 (m, 2H), 7.69-7.71 (m, 2H), 7.25 (s, 1H), 6.86 (s, 1H), 6.41 (br s, 2H), 5.94 (s, 2H), 4.17-4.20 (m, 2H), 3.65-3.67 (m, 2H), 1.73-1.75 (m, 2H), 1.62-1.65 (m, 2H), 1.36-1.38 (m, 4H); ^{13}C NMR (150 MHz, $CDCl_3/CD_3OD$) δ 168.62, 154.32, 152.37, 151.30, 149.61, 149.39, 147.26, 134.03, 132.07, 126.47, 123.26, 119.60, 119.46, 113.51, 102.55, 93.46, 43.80, 37.91, 29.54, 28.47, 26.45, 26.25; MS: *m/z* = 643.1 $[M + H]^+$.

9-(3-Aminohexyl)-8-(6-iodobenzo[d][1,3]dioxol-5-ylthio)-9H-purin-6-amine [3]. To a suspension of **6** (50 mg, 0.0778 mmol) in MeOH/CH_2Cl_2 (0.7:0.1 mL) was added hydrazine hydrate (66 μL, 68.4 mg, 1.375 mmol) and the mixture was stirred at rt for overnight. Solvent was removed under reduced pressure and the resulting residue was purified by preparatory TLC (CH_2Cl_2:MeOH-NH_3 (7N), 10:1) to give 30 mg (75%) of **7**. ^{1}H NMR (600 MHz, $CDCl_3$/ CD_3OD) δ 8.14 (s, 1H), 7.44 (s, 1H), 6.78 (s, 1H), 6.05 (s, 2H), 4.11-4.14 (m, 2H), 3.18 (m, 2H), 1.64-1.67 (m, 2H), 1.23-1.25 (m, 6H); ^{13}C NMR (150 MHz, $CDCl_3/CD_3OD$) δ 155.76, 153.53, 151.42, 149.31, 148.91, 144.26, 129.04, 119.98, 119.12, 111.33, 102.92, 91.19, 43.68, 41.85, 33.44, 29.61, 26.48, 26.39; MS: *m/z* = 513.1 $[M + H]^+$.

1-(6-((6-(6-amino-8-((6-iodobenzo[d][1,3]dioxol-5-yl)thio)-9H-purin-9-yl)hexyl)amino)-6-oxohexyl)-2-((E)-2-((E)-3-(2-((E)-3,3-dimethyl-5-sulfonato-1-(4-sulfonatobutyl)indolin-2-ylidene)ethylidene)-2-(4-sulfonatophenoxy)cyclohex-1-en-1-yl)vinyl)-3,3-dimethyl-3H-indol-1-ium-5-sulfonate [PU-800CW (4)]: To a solution of **3** (1 mg, 0.002 mmol) in DMF (0.1 mL) was added NIR Dye 800CW NHS Ester (3.5 mg, 0.003 mmol) and N,N-diisopropylethylamine (1 μL, 0.0057 mmol) and the reaction mixture was stirred at rt for overnight. Solvent was removed under reduced pressure to give 2.9 mg of **PU-800CW** (96%). LC-MS: (5%→95% B over 8 minutes; A= H_2O+0.1%TFA, B= CH_3CN+0.1%TFA), t_R = 2.80 min, Purity > 95%; MS: *m/z* = 1496.1, 747.6. ^{1}H NMR (600 MHz, DMSO-d_6) δ ^{1}H NMR (600 MHz, DMSO) δ 8.57 (s, 1H), 7.83 (t, *J* = 14.4 Hz, 2H), 7.77 (t, *J* = 5.5 Hz, 1H), 7.72-7.66 (m, 6H), 7.59 (s, 1H), 7.43 (d, *J* = 8.3 Hz, 1H), 7.33 (d, *J* = 8.4 Hz, 1H), 7.15 (d, *J* = 8.7 Hz, 2H), 7.11 (s, 1H), 6.31 (d, *J* = 14.2 Hz, 1H), 6.21

– 6.16 (m, 3H), 4.28 (t, *J* = 7.0 Hz, 2H), 4.21-4.15 (m, 4H), 3.02 (dd, *J* = 12.3, 6.3 Hz, 2H), 2.77-2.74 (m, 4H), 2.67 (t, *J* = 6.9 Hz, 2H), 2.06 (t, *J* = 7.2 Hz, 2H), 1.98 (m, 2H), 1.82 – 1.71 (m, 8H), 1.57 (dt, *J* = 14.6, 7.4 Hz, 2H), 1.37-1.29 (m, 20H); HRMS (m/z): $[M+3H+Na]^+$ calcd for $C_{64}H_{73}IN_8O_{16}NaS_5$ 1519.2651; found 1519.2691.

Synthesis of C-800CW

8-((2-Methoxyethyl)thio)-9H-purin-6-amine [6]: A mixture of 8-mercaptopurine (**5**; 441 mg, 2.64 mmol), 1-iodo-2-methoxyethane (573 mg, 3.08 mmol) and KOH (1.5 M (*aq.*), 2.2 mL) was stirred at rt for 24 h. Solvent was removed under reduced pressure and the residue purified by column chromatography (CH_2Cl_2:CH_3OH; 100:0 to 85:15) to yield 520 mg (87%) of **6**. ^1H NMR (500 MHz, DMSO-d_6) δ 13.01 (br s, 1H), 8.05 (s, 1H), 7.01 (br s, 2H), 3.62-3.64 (m, 2H), 3.44-3.46 (m, 2H), 3.28 (s, 3H); ^{13}C NMR (125 MHz, DMSO-d_6) δ 154.34, 152.64, 152.00, 147.20, 119.74, 71.00, 58.39, 31.10; MS (ESI) *m/z* 225.9 $[M+H]^+$.

2-(6-(6-amino-8-((2-methoxyethyl)thio)-9H-purin-9-yl)hexyl)isoindoline-1,3-dione [7]: To a solution of **6** (250 mg, 1.11 mmol) in DMF (2 mL) was added of Cs_2CO_3 (541 mg, 1.67 mmol) and N-(6-bromohexyl)-phthalimide (1.724 g, 5.55 mmol) of N-(6-bromohexyl)-phthalimide were added and the mixture was stirred at rt for 6 hr. Solvent was removed under reduced pressure and the resulting residue was purified by preparatory TLC (CH_2Cl_2:CH_3OH:CH_3COOH, 300:2:4) to yield 205 mg (38%) of **7**. ^1H NMR (600 MHz, $CDCl_3$/ CD_3OD) ^1H NMR (600 MHz, $CDCl_3$) δ 8.15 (s, 1H), 7.84-7.86 (m, 2H), 7.75-7.77 (m, 2H), 4.12 (t, *J* = 7.3 Hz, 2H), 3.76 (t, *J* = 6.1 Hz, 2H), 3.68 (t, *J* = 7.2 Hz, 2H), 3.52 (t, *J* = 6.1 Hz, 2H), 3.41 (s, 3H), 1.83-1.84 (m, 2H), 1.68-1.70 (m, 2H), 1.41-1.42 (m, 4H); ^{13}C NMR (150 MHz, $CDCl_3$/ CD_3OD) δ ^{13}C NMR (151 MHz, $CDCl_3$) δ 172.65, 157.49, 155.23, 155.06, 153.82, 138.02, 135.84, 127.08, 123.15, 74.45, 62.48, 47.02, 41.68, 35.58, 32.97, 32.24, 30.21, 29.97. MS (ESI) *m/z* 455.2 $[M+H]^+$.

9-(6-aminohexyl)-8-((2-methoxyethyl)thio)-9H-purin-6-amine [8]: To a solution of **7** (205 mg, 0.48 mmol) in MeOH/CH_2Cl_2 (0.7:0.1 mL) was added hydrazine hydrate (330 μL, 342 mg, 6.875 mmol) and the mixture was stirred at rt for overnight. Solvent was removed under reduced pressure and the resulting residue was purified by preparatory TLC (CH_2Cl_2:CH_3OH-NH_3 (7N); 10:1) to yield 95 mg (61%) of **8**. ^1H NMR (500 MHz, $CDCl_3$/CD3OD) δ 8.18 (s, 1H), 3.76 (t, *J* = 6.0 Hz, 2H), 3.52 (t, *J* = 6.0 Hz, 2H), 3.42 (s, 3H), 2.77-2.79 (m, 2H), 1.95 (br s, 2H), 1.81-1.82 (m, 2H), 1.57-1.58 (m, 2H), 1.39 (br s, 4H); ^{13}C NMR (125 MHz, $CDCl_3$) δ ^{13}C NMR (125 MHz, $CDCl_3$/CD3OD) δ 157.42, 155.34, 155.27, 153.74, 123.17, 74.43, 62.64, 46.92, 43.68, 35.63, 32.95, 32.88, 29.99, 29.91; MS (ESI) *m/z* 325.1 $[M+H]^+$.

1-(6-((6-(6-amino-8-((2-methoxyethyl)thio)-9H-purin-9-yl)hexyl)amino)-6-oxohexyl)-2-((E)-2-((E)-3-(2-((E)-3,3-dimethyl-5-sulfonato-1-(4-sulfonatobutyl)indolin-2-ylidene)ethylidene)-2-(4-sulfonatophenoxy)cyclohex-1-en-1-yl)vinyl)-3,3-dimethyl-3H-indol-1-ium-5-sulfonate [C-800CW (9)]: To a solution of **8** (1 mg, 0.003 mmol) in DMF (0.1 mL) was added NIR Dye 800CW NHS Ester (4.7 mg, 0.004 mmol) and N,N-diisopropylethylamine (1 μL, 0.0057 mmol) and the reaction mixture was stirred at rt for overnight. Solvent was removed under reduced pressure to give 2.9 mg of **C-800CW** (91%). LC-MS: (5%→95% B over 8 minutes; A= H_2O+0.1%TFA, B= CH_3CN+0.1%TFA), t_R = 2.55 min, Purity > 95%; MS (ESI) *m/z* 1310.9, 655.5. ^1H NMR (600 MHz, CD_3OD) δ 8.25 (s, 1H), 7.97 (d, J = 13.5 Hz, 1H), 7.93-7.78 (m, 7H), 7.36 (d, J = 7.7 Hz, 1H), 7.23 (d, J = 8.1 Hz, 1H), 7.17 (d, J = 11.8 Hz, 2H), 6.29 (d, J = 13.8 Hz, 1H), 6.15 (d, J = 13.6 Hz, 1H), 4.21 – 4.04 (m, 6H), 3.72 (t, J = 6.0 Hz, 2H), 3.59 (t, J = 6.0 Hz, 2H), 3.35 (s, 3H), 3.09 (t, J = 7.0 Hz, 2H), 2.92 (s, 2H), 2.82-2.69 (m, 4H), 2.14 (t, J = 7.2 Hz, 2H), 2.03 (s, 2H), 1.95 (s, 4H), 1.85-1.74 (m, 4H), 1.63 (p, J = 7.3 Hz, 2H), 1.45 – 1.27 (m, 20H).; HRMS (m/z): $[M+H+3Na]^+$ calcd for $C_{60}H_{74}IN_8O_{15}Na_3S_5$ 1375.3536; found 1375.3570.
