# Supplementary material for: Phosphorylation-driven epichaperome assembly is a regulator of cellular adaptability and proliferation
Source: Nat Commun. 2024 Oct 16;15:8912. doi: 10.1038/s41467-024-53178-5 (PMC11484706; doi:10.1038/s41467-024-53178-5)
Supplement: Supplementary file 2 — Description of Additional Supplementary Files [file 41467_2024_53178_MOESM2_ESM.pdf]

Description of Additional Supplementary Files

File name: Supplementary Data 1
Description: Contains LC-MS data and data analysis of PU-H71 and GA pulldown samples as well as 300 kDa band sliced from native-PAGE, associated with Figure 1d,1e, and Supplementary Figure 2c,3.

File name: Supplementary Data 2
Description: Contains LC-MS data and data analysis for the identification and quantitation of HSP90 cross-linked peptides in PU-H71 or GA pull-down samples, associated with Figure 2b.

File name: Supplementary Data 3
Description: Contains LC-MS data analysis of the HSP90 band from PU-H71 pulldown or lysate samples, associated with Figure 3b,3c.

File name: Supplementary Data 4
Description: Contains LC-MS data analysis for label-free quantitation of phosphopeptides from the HSP90 band of PUH71 or YK5-B pull-down samples from a variety of cell lines, associated with Figure 3d,3e.

File name: Supplementary Data 5
Description: Contains the RMSF data for the six individual pentameric assemblies, associated with Figure 5c and Supplementary Figure 8a-c.

File name: Supplementary Data 6
Description: Contains LC-MS data analysis for SILAC quantitation of phosphopeptides from WT mCherry-HSP90 in ES or differentiated trophoblast state, associated with Figure 7b.

File name: Supplementary Data 7
Description: Contains LC-MS data for SILAC quantitation of mCherry-HSP90 EE or AA mutant pull-down experiments in three replicates, associated with Figure 7c and Supplementary Figure 10b,10c. Normalized median intensity SILAC ratios were used for quantitation.
