# Supplementary material for: Phosphorylation-driven epichaperome assembly is a regulator of cellular adaptability and proliferation
Source: Nat Commun. 2024 Oct 16;15:8912. doi: 10.1038/s41467-024-53178-5 (PMC11484706; doi:10.1038/s41467-024-53178-5)
Supplement: Supplementary file 10 — Reporting Summary [file 41467_2024_53178_MOESM10_ESM.pdf]

nature portfolio

Corresponding author(s): Gabriela Chiosis and Feixia Chu

Last updated by author(s): Sep 23, 2024

# Reporting Summary

Nature Portfolio wishes to improve the reproducibility of the work that we publish. This form provides structure for consistency and transparency in reporting. For further information on Nature Portfolio policies, see our Editorial Policies and the Editorial Policy Checklist.

## Statistics

For all statistical analyses, confirm that the following items are present in the figure legend, table legend, main text, or Methods section.

| n/a | Confirmed | |
|---|---|---|
| ☐ | ☒ | The exact sample size (*n*) for each experimental group/condition, given as a discrete number and unit of measurement |
| ☐ | ☒ | A statement on whether measurements were taken from distinct samples or whether the same sample was measured repeatedly |
| ☐ | ☒ | The statistical test(s) used AND whether they are one- or two-sided<br>*Only common tests should be described solely by name; describe more complex techniques in the Methods section.* |
| ☒ | ☐ | A description of all covariates tested |
| ☐ | ☒ | A description of any assumptions or corrections, such as tests of normality and adjustment for multiple comparisons |
| ☐ | ☒ | A full description of the statistical parameters including central tendency (e.g. means) or other basic estimates (e.g. regression coefficient) AND variation (e.g. standard deviation) or associated estimates of uncertainty (e.g. confidence intervals) |
| ☐ | ☒ | For null hypothesis testing, the test statistic (e.g. *F*, *t*, *r*) with confidence intervals, effect sizes, degrees of freedom and *P* value noted<br>*Give P values as exact values whenever suitable.* |
| ☒ | ☐ | For Bayesian analysis, information on the choice of priors and Markov chain Monte Carlo settings |
| ☒ | ☐ | For hierarchical and complex designs, identification of the appropriate level for tests and full reporting of outcomes |
| ☒ | ☐ | Estimates of effect sizes (e.g. Cohen's *d*, Pearson's *r*), indicating how they were calculated |

*Our web collection on statistics for biologists contains articles on many of the points above.*

## Software and code

Policy information about availability of computer code

Data collection

As described in the Methods, the identity and purity of each product was characterized by MS, HPLC, TLC, and NMR. 1H/13C NMR spectra were recorded on either a Bruker 500 or 600 MHz instrument (Bruker, Billerica, MA, USA). High resolution mass spectra were recorded on a Waters LCT Premier system (Waters Corporation, Milford, CT, USA). Low resolution mass spectra were obtained on Waters Acquity Ultra-Performance LC with electrospray ionization and SQ detector (Waters Corporation, Milford, CT, USA). HPLC analysis was done on Waters Autopurification system with PDA, MicroMass ZQ and ELSD detector and a reversed phase column (Waters X-Bridge C18, 4.6 x 150 mm, 5 µm) eluted with water/acetonitrile gradients, containing 0.1% TFA. The chemiluminescent signal was detected with Enhanced Chemiluminescence (ECL) reagent according to manufacturer's instructions and visualized using Biorad ChemiDoc MP imaging systems (Bio-Rad Laboratories, Hercules, CA, USA) and analyzed using Image Studio Lite version 5.2 (LI-COR Biosciences, Lincoln, NE, USA). For PUTCO in-gel fluorescence, Biorad ChemiDoc MP imaging systems (Bio-Rad Laboratories, Hercules, CA, USA) instrument was used and images were analyzed using Image Lab 6.1 (Bio-Rad Laboratories, Hercules, CA, USA) . For confocal microscopy, fluorescence was visualized under a Leica SP8 Stellaris microscope (Leica Microsystems, Wetzlar, Germany) and images were analyzed using Image J (version 1.54f) and Leica LAS X lite (version 2.6.0) software. For PU-CW800 in-gel fluorescence, LI-COR Odyssey CLx instrument (LI-COR Biosciences, Lincoln, NE, USA) was used and images were analyzed using Image StudioTM software (LI-COR Biosciences, v5.2) . Luminescence was measured using a Perkin Elmer EnVision 2104 Multi-label Plate Reader (Perkin Elmer, Waltham, MA, USA). For quantitative proteomics, samples were analyzed by LC-MS/MS with a Q Exactive High Field Orbitrap (Thermo Scientific, Waltham, MA, USA), and resulting spectra searched with MaxQuant using its corresponding TMT label as variable modifications on N-terminus and lysine. Label free proteomics analyses were performed using a Q Exactive mass spectrometer coupled to a Thermo Scientific EASY-nLC 1000. The mass spectrometer was operated in data-dependent analysis (DDA) mode with survey scans acquired at a resolution of 70,000 over a scan range of 300-1750 m/z. Cross-linking mass spectrometry data were recorded by LTQ Orbitrap XL mass spectrometer (Thermo Scientific, Waltham, MA, USA). LC-MS data were acquired in a data-dependent acquisition mode, cycling between a MS scan (m/z 315–2,000) acquired in the Orbitrap, followed by low-energy CID analysis on three most intense multiply charged precursors acquired in the linear ion trap. Molecular dynamics (MD) simulations were conducted using the Schrödinger Suite

release 2022-3. Modeller v10.4 was used to prepare protein structures for the HSP90 assemblies based on cryo-EM structures, and phosphorylated states were modeled accordingly. Each simulation was run thrice for 100 ns, and the resulting trajectories were saved for subsequent analysis. IUPred web server was used to predict disorder regions (PMID: 15955779).

Data analysis

Statistical analyses were conducted using Prism (version 9, GraphPad Software)(t-tests and ANOVA).
ImageJ version 1.54f was used for western blot quantification.
In cross-linking mass spectrometry, the centroided peak lists of the CID spectra were generated using PAVA searched against a database that is consisted of the Swiss-Prot protein database using Batch-Tag, a program of the University of California San Francisco Protein Prospector software, version 5.9.2. Cross-linked peptides were identified using an integrated module in Protein Prospector, based on a bioinformation strategy developed in the UCSF Mass Spectrometry Facility (PMIDs: 28971800; 29857191; 19809093; 25149264).
Structural illustrations were prepared using the UCSF Chimera (version 1.18) or PyMOL v2.3.0.
For protein identification by MS, all mass spectra were first converted to mgf peak list format using Proteome Discoverer 1.4 and the resulting mgf files searched against a human Uniprot protein database using Mascot. The Mascot search result was finally imported into Scaffold (Proteome Software, Inc., Portland, OR; version 4.11) to further analyze tandem mass spectrometry (MS/MS) based protein and peptide identifications. X! Tandem (The GPM, thegpm.org; version CYCLONE (2010.12.01.1) was then performed and its results were merged with those from Mascot. The mass spectra files were also subjected to Label-Free Quantitation (LFQ) using MaxQuant proteomics data analysis workflow (version 1.6.0.1) with the Andromeda search engine. For TMT experiments, all data were analyzed with the MaxQuant proteomics data analysis workflow (version 1.5.5.1) with the Andromeda search engine. Following MaxQuant analysis, the protein and peptide .txt files were imported into Perseus (version 1.5.6.0) software which was used for the statistical analysis of all the proteins identified.
Analysis of the MD simulation data was performed using the Desmond module in Schrödinger suite. All data were processed and visualized using Schrödinger's analysis tools, along with custom Python scripts where necessary.

For manuscripts utilizing custom algorithms or software that are central to the research but not yet described in published literature, software must be made available to editors and reviewers. We strongly encourage code deposition in a community repository (e.g. GitHub). See the Nature Portfolio guidelines for submitting code & software for further information.

# Data

Policy information about availability of data

All manuscripts must include a data availability statement. This statement should provide the following information, where applicable:

- Accession codes, unique identifiers, or web links for publicly available datasets
- A description of any restrictions on data availability
- For clinical datasets or third party data, please ensure that the statement adheres to our policy

The source data underlying all main and supplementary figures – raw data, statistical analyses and uncropped gels - are provided with this paper as a Source Data file and were deposited in the Figshare repository [https://doi.org/10.6084/m6089.figshare.27075415]113. Datasets and analytics associated with epichaperomics and proteomics analyses are available in the Supplementary Information as Supplementary Data 1 through 7 and were deposited in the figshare repository [https://doi.org/10.6084/m6089.figshare.26662333]114. LC-MS data (i.e., proteomics and epichaperomics raw mass spectrometry data, peak lists, and results) that support the findings of this study are deposited to the ProteomeXchange Consortium via the PRIDE partner repository with the dataset identifier PXD050251 [http://proteomecentral.proteomexchange.org/cgi/GetDataset?ID=PXD050251]. Protein sequences (FASTA files) were obtained from UniProt (https://www.uniprot.org/). MD simulations data were deposited in Zenodo entry 10800912 [https://doi.org/10.5281/zenodo.10800912]115. Source data are provided with this paper.

# Research involving human participants, their data, or biological material

Policy information about studies with human participants or human data. See also policy information about sex, gender (identity/presentation), and sexual orientation and race, ethnicity and racism.

Reporting on sex and gender

Reporting procedure was not part of this study. The source of samples consists of de-identified unused portions of surgical specimens that were taken for reasons other than research (i.e. for breast cancer patients undergoing the procedures for medical reasons unrelated to need for research samples or to the nature of the research). No individuals were excluded on the basis of age, sex, gender or ethnicity. Because breast cancer is a disease which overwhelmingly affects women, and is a disease that is generally not seen in children, the vast majority of patients enrolled were females >18yrs of age. The patient sex and gender was determined based on self-reporting. In the case of pancreatic cancer samples (n = 3 patients), sex information was not available. An ethnically and socioeconomically diverse population is likely represented in the study as anticipated based on the New York demographics. Informed consent and HIPAA compliance forms were obtained from all subjects prior to their enrollment. Samples were de-identified before receipt for use in the studies.

Reporting on race, ethnicity, or other socially relevant groupings

See above.

Population characteristics

See above. Because breast cancer is a disease which overwhelmingly affects women, and is a disease that is generally not seen in children, the vast majority of patients enrolled were females >18yrs of age.

Recruitment

Recruitment procedure was not part of this study (see also above). No compensation was provided for participation in this research.

Ethics oversight

Surgical specimens were obtained in accordance with the guidelines and approval of the Institutional Review Board at Memorial Sloan Kettering Cancer Center, Biospecimen Research Protocol# 09-121, project title: Ex-Vivo Testing of Breast Cancer Tumors for Sensitivity to Inhibitors of Heat Shock Proteins and Signaling Pathway Inhibitors, S. Modi, PI, and Biospecimen Research Protocol# 14-091, project title: Establishment and Characterization of Unique Mouse Models Using Patient-Derived Xenografts . E. de Stanchina, PI.

Note that full information on the approval of the study protocol must also be provided in the manuscript.

# Field-specific reporting

Please select the one below that is the best fit for your research. If you are not sure, read the appropriate sections before making your selection.

☒ Life sciences ☐ Behavioural & social sciences ☐ Ecological, evolutionary & environmental sciences

For a reference copy of the document with all sections, see nature.com/documents/nr-reporting-summary-flat.pdf

# Life sciences study design

All studies must disclose on these points even when the disclosure is negative.

| | |
|---|---|
| Sample size | No formal statistical methods were used to predetermine sample sizes for in vitro, biochemical, or functional validation experiments. However, sample sizes were selected based on common practices in the field, taking into consideration the variability and reproducibility of similar assays in previously published studies. For cell-based experiments and biochemical assays, we typically used n = 3 or more biological replicates, which is standard for ensuring reproducibility and reliability of results. For experiments such as molecular dynamics simulations and crosslinking analyses, a minimum of three independent replicates were performed to provide robust data. The sample sizes for each experiment are fully disclosed in the manuscript, and were considered sufficient to draw valid conclusions based on the experimental aims. For the patient tissue samples, we selected n = 9 patients with paired tumor and tumor-adjacent tissues. This sample size was chosen based on availability of paired samples, as well as to account for biological variability between patients. Paired analysis allows for a more direct comparison between tumor and non-tumor tissues within the same patient, reducing inter-patient variability. Similar sample sizes are commonly employed in studies of this type, ensuring that the data are representative while still allowing for robust statistical analysis. The sample size was considered sufficient to identify significant trends while balancing feasibility and ethical considerations regarding patient tissue availability. |
| Data exclusions | No data were excluded from the analyses. |
| Replication | All in vitro experiments were performed in at least 3 biological replicates, with biological and technical replicates which are fully disclosed in the manuscript. Several alternative methods were used to validate observations. Experiments were also replicated through multiple cohort analyses. Results shown are representative of several independently performed experiments (see figure legends, at least 3). There were no findings that could not be replicated or reproduced. |
| Randomization | For in vitro experiments, samples were allocated into experimental groups by randomization. |
| Blinding | Investigators were not blinded to group allocation during data collection and/or analysis for the preclinical studies. Blinding was not relevant to the experiments described in the study due to primary investigators performing experiments from start to finish due to technicality required. |

# Reporting for specific materials, systems and methods

We require information from authors about some types of materials, experimental systems and methods used in many studies. Here, indicate whether each material, system or method listed is relevant to your study. If you are not sure if a list item applies to your research, read the appropriate section before selecting a response.

## Materials & experimental systems

| n/a | Involved in the study | |
|---|---|---|
| ☐ | ☒ | Antibodies |
| ☐ | ☒ | Eukaryotic cell lines |
| ☒ | ☐ | Palaeontology and archaeology |
| ☒ | ☐ | Animals and other organisms |
| ☒ | ☐ | Clinical data |
| ☒ | ☐ | Dual use research of concern |
| ☒ | ☐ | Plants |

## Methods

| n/a | Involved in the study | |
|---|---|---|
| ☒ | ☐ | ChIP-seq |
| ☒ | ☐ | Flow cytometry |
| ☒ | ☐ | MRI-based neuroimaging |

## Antibodies

| | |
|---|---|
| Antibodies used | All antibodies and relevant information is provided in the Methods and are also listed below:<br>β-actin (A1978, Sigma-Aldrich, RRID: AB_476692, 1:3000) was used as protein loading controls.<br>Primary antibodies used in this study are listed below:<br>HSP90β (SMC-107; RRID:AB_854214; 1:2,000) and HSP110 (SPC-195; RRID:AB_2119373; 1:1,000) from Stressmarq; HSC70 (SPA-815; RRID:AB_10617277; 1:1,000), HSP70 (ADI-SPA-810, RRID:AB_10616513, 1:2,000), and HOP (SRA-1500; RRID:AB_10618972; 1:1,000) from Enzo; HSP90α (ab2928; RRID:AB_303423; 1:6,000), AHA1 (ab56721, RRID:AB_2273725, 1:1,000) and anti-HA tag (ab9110, RRID:AB_307019; 1:1,000) from Abcam; p-MEK1/2 (S217/221) (9154; RRID:AB_2138017; 1:1,000), MEK1/2 (9122; RRID:AB_823567; 1:1,000), p-mTOR (S2448) (5536; RRID:AB_10691552; 1:500), mTOR (2983; RRID:AB_2105622; 1:1,000), CDC37 (4793; |

RRID:AB_10695539; 1:1,000), HOP (5670; RRID:AB_10828378; 1:1,000), p-S6 ribosomal protein (Ser235/236) (4858; RRID:AB_916156; 1:2,000), S6 ribosomal protein (2217; RRID:AB_331355; 1:3,000), Oct4 (2840, RRID:AB_2167691, 1:2,000), p-AKT (S473) (9271, RRID:AB_329825, 1:2,000), AKT (4691, RRID:AB_915783, 1:3000), CK2α (Cat# 2656, RRID: AB_2236816, 1:2,000) from Cell Signaling Technologies, β-actin (A1978, RRID: AB_476692, 1:3000) from Sigma-Aldrich, and mCherry (PA5-34974, RRID:AB_2552323, 1:2,000) and p-Ser226 HSP90β (PA5-105480, RRID:AB_2816908, 1:1,000) from Fisher Scientific.
HRP-conjugated secondary antibodies: goat anti-mouse (1030-05, RRID: AB_2619742, 1;5,000), goat anti-rabbit (4010-05, RRID: AB_2632593, 1:5,000) and goat anti-rat (3030-05, RRID: AB_2716837, 1:5,000) (Southern Biotech, Birmingham, AL, USA).

Validation

All antibodies are commercially available and have been validated by the manufacturer. Supporting publications are found on the manufacturer's site. Antibodies have been validated either from prior reports and studies or validated by the manufacturer as stated on the website from the catalog numbers listed above or published references on the manufacturers' websites. Manufacturer states the antibody has been validated for intended use. Manufacturer citation are listed in manufacturer website for each specific antibody.
Specifically, for signaling markers see Cell Signaling website for: p-MEK1/2 (S217/221) (9154; RRID:AB_2138017; 1:1,000), MEK1/2 (9122; RRID:AB_823567; 1:1,000): Western blot analysis of extracts from untreated or TPA-treated HeLa and NIH/3T3 cells, using Phospho-MEK1/2 (Ser217/221) (41G9) or MEK1/2 Antibody #9122 . p-mTOR (S2448) (5536; RRID:AB_10691552; 1:500), mTOR (2983; RRID:AB_2105622; 1:1,000): Western blot analysis of extracts from serum-starved NIH/3T3 cells, untreated or insulin-treated (150 nM, 5 minutes), alone or in combination with λ-phosphatase, using Phospho-mTOR (Ser2448) or mTOR (7C10) Rabbit mAb #2983. p-S6 ribosomal protein (Ser235/236) (4858; RRID:AB_916156; 1:2,000), S6 ribosomal protein (2217; RRID:AB_331355; 1:3,000): Western blot analysis of extracts from serum-starved MCF7 cells, untreated (-) or treated (+) with combinations of the following treatments as indicated: human IGF-1 (100 ng/mL, 10 min) and λ phosphatase, using Phospho-S6 Ribosomal Protein (Ser235/236) (D57.2.2E) XP® Rabbit mAb or S6 Ribosomal Protein (5G10) Rabbit mAb #2217. p-AKT (S473) (9271, RRID:AB_329825, 1:2,000), AKT (4691, RRID:AB_915783, 1:3000): Western blot analysis of extracts from NIH/3T3 cells, untreated or treated with PDGF for the indicated times, using Phospho-Akt (Ser473) or Akt antibody. CK2α (2656, RRID: AB_2236816, 1:2,000): Western blot analysis of HAP1 extracts from WT (left) or CSNK2A1 KO (right) using CK2α Antibody.
For pluripotency markers Oct4 (2840, RRID:AB_2167691, 1:2,000) see ESCs and differentiated ESCs (for ex. Supplementary Fig. 1).
For p-Ser226 HSP90β (PA5-105480, RRID:AB_2816908, 1:1,000) see ThermoFisher website: Western blot analysis of Phospho-HSP90 (Ser226) in HeLa cells treated with TNF-alpha, treated with phospho-blocking peptide; and treated with non-phospho-blocking peptide.
For anti tag antibodies (mCherry, HA): Western blot was performed by loading whole cell extracts of untransfected and transiently transfected lysates (see Figures 6,9, S Fig 13).
For epichaperome component detection and chaperone level quantification by HSP90β (SMC-107), HSP110 (SPC-195), HSC70 (SPA-815), HOP (SRA-1500), HSP90α (ab2928), AHA1 (ab56721), CDC37 (4793), HOP (5670), HSP70 (ADI-SPA-810) refer to siRNA and depletion experiments in prior publications (PMID: 27706135; PMID: 34824367) as well as the use of cell lines differentiated by epichaperome levels (i.e., epichaperome high, low and negative) for further validation (see prior publications such as PMID: 37540435, PMID: 27706135; PMID: 34824367; PMID: 37353488; PMID: 31949159; PMID: 30341316). Also, relevant positive and negative controls were used to further validate several antibodies as indicated in the figures (see the use of the well-characterized cell lines MDA-MB-468 and ASPC1 throughout the figures).
For β-actin (A1978, RRID: AB_476692, 1:3000) see Sigma website https://www.sigmaaldrich.com/US/en/search/a1978?focus=papers&page=1&perpage=30&sort=relevance&term=A1978&type=citation_search

## Eukaryotic cell lines

Policy information about cell lines and Sex and Gender in Research

Cell line source(s)

The MDA-MB-468 (female, breast cancer cell line, HTB-132, RRID: CVCL_0419), ASPC1 (female, pancreatic cancer cell line, CRL-1682, RRID: CVCL_0152), NCI-H1975 (female, non-small cell lung cancer cell line, CRL-5908, RRID: CVCL_1511), Daudi (male, B lymphoblast cell line, CCL-213, RRID: CVCL_0008), MRC5 (male, lung fibroblast cell line, CCL-171, RRID:CVCL_0440), CCD-18Co (female, colon fibroblast cell line, CRL-1459, RRID: CVCL_2379) and the Human Embryonic Kidney 293 (HEK293) cell line (CRL-1573, RRID: CVCL_0045), of female origin as determined by sequencing, were purchased from ATCC. IBL-1 (RRID:CVCL_9638) was derived from a male AIDS-related immunoblastic lymphoma patient93. Human mammary epithelial cells HMEC (PCS-600-010) isolated from adult female breast tissue were purchased from Lonza. B-cell lymphoma cell line OCI-LY1 (RRID:CVCL_1879), of male origin as determined by sequencing, was obtained from the Ontario Cancer Institute. E14 mouse ES cells94 were received as frozen ampules from TG Fazzio (U Mass Med School). Cells were feed-free and verified as of male mouse origin through sequencing. ZHBTc4 mouse ES cells derived from a male mouse31 were received from D. Levasseur (U of Iowa). Cells were cultured as ESCs without feeder cells in the absence of doxycycline. hiPSC were a gift from the Studer lab (MSKCC) and derived from fibroblasts from a healthy male donor, purchased from Coriell (#AG16146) and reprogrammed using CytoTune Sendai viruses34.

Authentication

Cell were authenticated using short tandem repeat profiling.

Mycoplasma contamination

Cells were routinely tested for mycoplasma and were found to be negative.

Commonly misidentified lines
(See ICLAC register)

No commonly misidentified cell lines were used.

## Plants

| | |
|---|---|
| Seed stocks | Does not apply. |
| Novel plant genotypes | Does not apply. |
| Authentication | Does not apply. |
